# The Seascape of Demersal Fish Nursery Areas in the North Mediterranean Sea, a First Step Towards the Implementation of Spatial Planning for Trawl Fisheries

**DOI:** 10.1371/journal.pone.0119590

**Published:** 2015-03-18

**Authors:** Francesco Colloca, Germana Garofalo, Isabella Bitetto, Maria Teresa Facchini, Fabio Grati, Angela Martiradonna, Gianluca Mastrantonio, Nikolaos Nikolioudakis, Francesc Ordinas, Giuseppe Scarcella, George Tserpes, M. Pilar Tugores, Vasilis Valavanis, Roberto Carlucci, Fabio Fiorentino, Maria C. Follesa, Magdalena Iglesias, Leyla Knittweis, Eugenia Lefkaditou, Giuseppe Lembo, Chiara Manfredi, Enric Massutí, Marie Louise Pace, Nadia Papadopoulou, Paolo Sartor, Christopher J. Smith, Maria Teresa Spedicato

**Affiliations:** 1 Istituto per l’Ambiente Marino Costiero (IAMC), Consiglio Nazionale delle Ricerche, Mazara del Vallo, Italy; 2 COISPA Tecnologia & Ricerca, Bari, Italy; 3 Istituto di Scienze Marine (ISMAR), Consiglio Nazionale delle Ricerche, Ancona, Italy; 4 Department of Economics, Roma Tre University, Rome, Italy; 5 Hellenic Centre for Marine Research, Heraklion, Greece; 6 Instituto Español de Oceanografía, Centre Oceanogràfic de les Balears, Palma de Mallorca, Spain; 7 Department of Biology, University of Bari, Bari, Italy; 8 Department of Life and Environmental Sciences, University of Cagliari, Cagliari, Italy; 9 Department of Biology, University of Malta, Msida, Malta; 10 Laboratorio Biologia Marina Fano, Università di Bologna, Bologna, Italy; 11 Department of Fisheries and Aquaculture, Ministry for Sustainable Development, the Environment and Climate Change, Santa Venera, Malta; 12 Centro Interuniversitario di Biologia Marina ed Ecologia Applicata “G. Bacci” (CIBM), Livorno, Italy; Technical University of Denmark, DENMARK

## Abstract

The identification of nursery grounds and other essential fish habitats of exploited stocks is a key requirement for the development of spatial conservation planning aimed at reducing the adverse impact of fishing on the exploited populations and ecosystems. The reduction in juvenile mortality is particularly relevant in the Mediterranean and is considered as one of the main prerequisites for the future sustainability of trawl fisheries. The distribution of nursery areas of 11 important commercial species of demersal fish and shellfish was analysed in the European Union Mediterranean waters using time series of bottom trawl survey data with the aim of identifying the most persistent recruitment areas. A high interspecific spatial overlap between nursery areas was mainly found along the shelf break of many different sectors of the Northern Mediterranean indicating a high potential for the implementation of conservation measures. Overlap of the nursery grounds with existing spatial fisheries management measures and trawl fisheries restricted areas was also investigated. Spatial analyses revealed considerable variation depending on species and associated habitat/depth preferences with increased protection seen in coastal nurseries and minimal protection seen for deeper nurseries (e.g. *Parapenaeus longirostris* 6%). This is partly attributed to existing environmental policy instruments (e.g. Habitats Directive and Mediterranean Regulation EC 1967/2006) aiming at minimising impacts on coastal priority habitats such as seagrass, coralligenous and maerl beds. The new knowledge on the distribution and persistence of demersal nurseries provided in this study can support the application of spatial conservation measures, such as the designation of no-take Marine Protected Areas in EU Mediterranean waters and their inclusion in a conservation network. The establishment of no-take zones will be consistent with the objectives of the Common Fisheries Policy applying the ecosystem approach to fisheries management and with the requirements of the Marine Strategy Framework Directive to maintain or achieve seafloor integrity and good environmental status.

## Introduction

Understanding the patterns of spatial distribution of wild populations is a critical consideration in the development of effective management and conservation strategies [1]. For marine populations of highly mobile species, such as fish and shellfish, this exercise can be complicated by the relevant shifts in habitat preferences that individuals display during their life. Changes related to body size in foraging, spawning cycles and habitat preferences lead to the displacement of different groups of individuals in different patches of the environmental mosaic. The spatial distribution of many marine populations can consequently be depicted as a mosaic of habitat patches that are functionally connected at multidimensional levels [2]. This type of ecological knowledge is becoming increasingly important to help achieve a sustainable exploitation of commercially important marine populations through the protection of critical habitats (i.e. Essential Fish Habitat: EFH), which play a key role for population processes such as spawning and recruitment.

In the Mediterranean Sea, a semi-enclosed highly biodiverse basin, where more than 90% of the harvested stocks are overexploited mostly due to a un-selective exploitation pattern [3, 4] and opportunistic fishery behaviour [5], the protection of the main nurseries of commercial species is increasingly viewed as a major step toward the achievement of more sustainable exploitation patterns. The current compliance of Mediterranean trawl fisheries with minimum conservation reference sizes (MCRS) is very low for some stocks such as hake (*Merluccius merluccius*) and the deep-water rose shrimp (*Parapenaeus longirostris*). Due to poor enforcement and reduced trawl selectivity, some sectors show up to 70% of the catch of specimens below the MCRS [4]. Such an exploitation pattern is hindering the achievement of Maximum Sustainable Yield (MSY) for fisheries, as required both by the new European Common Fisheries Policy (CFP) and the EU Marine Strategy Framework Directive (MSFD), as well as the maximisation of fleet revenues [3], [4]. The current annual amount of discards from the Mediterranean fisheries has been estimated to be over 200.000 t, corresponding to a percentage of 13–27% of the overall catch, with a proportion of up to 60% for trawl fisheries [6].

The recent landing obligation, introduced by the reform of the CFP (EU Reg. N. 1380/2013), is promoting the application of technical measures aimed at discouraging the capture of undersized specimens of commercial species. These measures include the spatial closure of areas where juveniles congregate at the end of their planktonic dispersal phase, often occurring in fragile habitats such as seagrass meadows [7], [8], *Cystoseira* forests [9], *Peyssonnelia* beds [10] and crinoid beds [11].

The Council Regulation EC 1967/2006 provides some guidelines for the implementation of habitat conservation in the Mediterranean Sea, with particular attention devoted to the protection of nursery areas. This regulation has the potential to yield important conservation benefits and it is primarily based on two assumptions: (1) juvenile fish are particularly vulnerable to a fine mesh trawl fishery [12], especially when they are concentrated in nursery areas, and (2) a reduction of fishing mortality on immature fish represents a fundamental prerequisite for sustainable fisheries in the direction of the “spawn at least once” rule [13].

In the last 20 years, a line of research has focused on identifying the most important nursery habitats based on their contribution to adult populations [14, 15] inter alia by developing different metrics [16, 17] and considering a temporal component [18]. Recently, Nagelkerken et al., [2] have provided a valuable step forward in the process leading to the identification and management of critical nursery areas by focusing on the identification of highly productive patches, especially in situations where large habitat units cannot be protected as a whole due to socio-economic, practical or other considerations. This is a common situation in the Mediterranean Sea, where nurseries of commercial demersal species often are distributed in offshore habitats such as the shelf break, covering large extensions of the sea bottom. Overall the most common approach used to identify nurseries was based on fish density measures applied as indices of recruitment importance [19–28]. However, most of the studies carried out so far were generally conducted over rather small areas, covering restricted time periods, and using different approaches, which make comparisons at larger scales unfeasible.

In order to expand the analysis to a larger spatio-temporal scale we analysed the spatial distribution of nurseries of some of the main commercial species of demersal fish and shellfish exploited in the EU Mediterranean basin, using the longest existing time series of standardized abundance data collected on demersal fish and shellfish in the North Mediterranean Sea (i.e MEDITS trawl survey, [29]) as one of the main outcomes from the EU MEDISEH (Mediterranean Sensitive Habitats) research project (http://mareaproject.net/). Annual nurseries were identified by modelling the spatial distribution of recruits with the purpose of reconstructing the distribution of “density hot-spots” (i.e. areas of higher density), regarded as more productive areas. As a second step, each annual density hot-spot was ranked according to its temporal persistence throughout the study period. Our main assumption was that the average contribution to the adult population could be expected to be higher from nurseries with higher densities of recruits and higher spatio-temporal stability. In fact, the maintenance of a population mostly depends on the success of the displacement of young fish into nursery areas and their recruitment from nursery areas to the parental population [30]. The location of nursery areas is therefore an integral component of the adaptation of marine fish life cycles to their environment. In this context, the stability of a density hot-spot of recruits in a given area can be assumed to be indirect evidence of the importance of that area for the recruitment/spawning success of the population. Furthermore, the temporal persistence of the characteristics of an area is a fundamental prerequisite for its inclusion in a conservation network, as commonly considered in terrestrial ecosystems [31]. Based on these assumptions, we adopted a 6-step approach to identify and classify nursery areas of commercial species for conservation purposes.

In particular, we (1) defined the proportion of the population corresponding to the recruits; (2) estimated the distribution of recruits’ densities using several modelling approaches depending on species and area; (3) identified density hot-spots using a standardized procedure; (4) provided measures of persistence of each identified hot-spot; (5) evaluated the importance of each area in a multispecies context by analysing the degree of overlap among nursery areas within the set of species considered. Finally, (6) we calculated the spatial overlap between nursery areas and the current fisheries restricted areas as a basis for the implementation of spatial conservation planning for demersal fisheries resources in the North Mediterranean Sea.

## Material and Methods

A total of 11 demersal species were included in the analysis: 3 crustaceans (giant red shrimp: *Aristaeomorpha foliacea*, Norway lobster: *Nephrops norvegicus*, deep-water rose shrimp: *Parapenaeus longirostris*), 2 cephalopods (broadtail shortfin squid: *Illex coindetii*, horned octopus: *Eledone cirrhosa*) and 6 fish (European hake: *Merluccius merluccius*, red mullet: *Mullus barbatus*, common Pandora: *Pagellus erythrinus*, common sole: *Solea solea*, blackmouth catshark: *Galeus melastomus*, thornback ray: *Raja clavata)* distributed either on the continental shelf or upper slope, between 10 and 800 m depth (Table 1).

**Table 1:** List of the species and Mediterranean geographical sub-areas (GSAs) where recruit density was modelled for nursery identification.

	GSAs											
Species	1–6	5	7	9	10	11	15–16	17	18	19	20	22–23
*M*. *merluccius*	Z	Z	GAMM	GAMM	Z+OK	Z+OK	GAMM	Z	Z+OK	Z+OK	Z+IDW	Z+IDW
*M*. *barbatus*								Z	Z			
*P*. *erythrinus*					OK	OK		Z	OK			
*R*. *clavata*						Z+OK	GLMM					
*G*. *melastomus*	Z	Z	GAMM	INLA	Z+OK	Z+OK	GLMM		Z+OK	Z+OK		
*S*. *solea*								OK				
*A*. *foliacea*					Z+OK	Z+OK	GAMM		Z+OK	Z+OK		
*P*. *longirostris*	Z			INLA	Z+OK	Z+OK	GLMM	Z	OK	Z+OK		Z+IDW
*N*. *norvegicus*						OK	GLMM	Z		OK		
*E*. *cirrhosa*			GAMM	GAMM	Z+OK	Z+OK		Z	Z+OK	Z+IDW		Z+IDW
*I*. *coindetii*	Z			GAMM	Z+OK	Z+OK	GAMM	OK	Z+OK	Z+OK	Z+IDW	Z+IDW

The modelling approaches used are indicated: GLMM (generalized linear mixed models); GAMM (generalized additive mixed models); INLA (Bayesian GLMM); Z (zero inflated generalized additive models, ZIGAM); OK (ordinary kriging); IDW (interpolate distance weighting)

**Fig 1:**
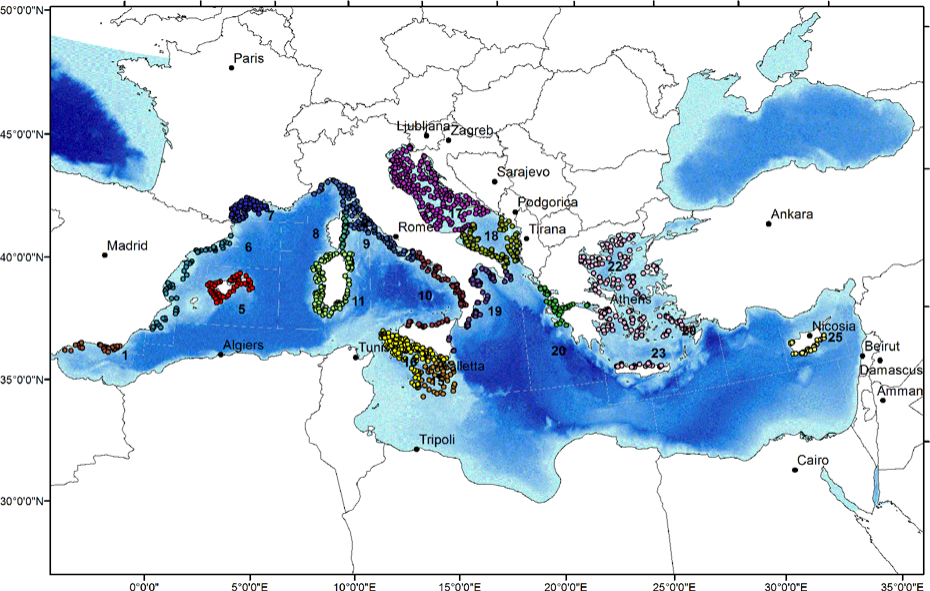
Map showing the study area with the position of the MEDITS trawl stations (year 2008). Numbers refer to Mediterranean FAO-GFCM GSAs (Geographical Sub-Areas). 1 Northern Alboran Sea, 5 Balearic Islands, 6 Northern Spain, 7 Gulf of Lions, 8 Corsica Island, 9 Ligurian and North Tyrrhenian Sea, 10 South Tyrrhenian Sea, 11 Sardinia, 15 Malta Island, 16 South of Sicily, 17 Northern Adriatic, 18 Southern Adriatic Sea, 19 Western Ionian Sea, 20 Eastern Ionian Sea, 22 Aegean Sea, 23 Crete Island, 25 Cyprus Island.

The species included in our study were selected from amongst the most commercially important Mediterranean demersal species of fish and shellfish, mostly exploited by trawl fisheries in a wide depth range, from the coastal shelf (e.g. red mullet, common sole, common Pandora) and outer shelf (e.g. European hake, horned octopus, deep-water rose shrimp) to the continental slope (e.g. blackmouth catshark, thornback ray, giant red shrimp, Norway lobster). The spatial scale adopted for the identification of nurseries was the FAO-GFCM (Food and Agriculture Organisation—General Fisheries Commission for the Mediterranean) Geographical Sub-Areas (GSAs, Fig. 1). These are currently recognized as management areas by both the GFCM and the EU assuming that, in the lack of knowledge on the spatial population structure of the main commercial species, separate stock units occur in each GSA. As a consequence, the ongoing spatial stratification for data collection (EU-DCF, Commission Decision 2010/93/EU and GFCM Task 1 Statistical Matrix) is based on the GSA subdivision of the Mediterranean Sea. Taking into account such assumptions, we separately analysed the distribution of recruits for each GSA, with the exception of GSA 1—GSA 6 (Northern Alboran Sea and Northern Spain), GSA 15—GSA 16 (Maltese Islands and South of Sicily) and GSA 22—GSA 23 (Aegean Sea and Crete Island) whose data were combined for the analyses. In these areas we assumed the existence of shared units of stocks based on the knowledge of the fisheries biologists involved in the study.

Analyses were based on the MEDITS bottom trawl survey data [29] for the period 1994–2010 (see Fig. 1) with the only exception of common sole in Northern Adriatic Sea, modelled using the SoleMon beam trawl survey data, available from 2005 to 2010 [32].

MEDITS is carried out in EU Mediterranean waters in late spring (May-June) to gather data on benthic and demersal fish and shellfish in a wide depth range, from 10 to 800 m depth. The survey adopts a standardized trawl net (GOC 73) with vertical opening of 2.5–3 m and a very fine stretched mesh (20-mm) in the cod-end. The net was therefore designed to catch the wide range of species characterised within Mediterranean demersal communities, from strictly benthic to bentho-pelagic organisms, in a large range of sizes, including small-sized juveniles [29]. In GSAs 17 (Northern Adriatic) a shorter time series was used because MEDITS data for Croatian and Slovenian waters have only been available since 2002 and therefore data for the years 1994–2001 refer only to the Italian side. Similarly, in GSAs 15–16 data from Maltese waters were available from 2003. Thus, to obtain a global picture of the distribution of nursery areas in GSAs 15–16 and 17 we excluded the years where only a portion of the GSAs was covered. In GSA 5 (Balearic Islands), the MEDITS trawl survey started in 2007.

### Recruits identification

The first step in our analytical framework was the identification of recruits in the sampled population. Recruits were considered as those specimens that have settled on the bottom, becoming available to the fishing gear in well-defined habitats at the end of their larval—pelagic stage and which remain in these habitats before dispersing or migrating. Due to the lack of specific studies on the dispersal behaviour of juveniles (see for example [33]), we assumed that the first separable modal component of the age group 0 was composed of specimens recently settled on the bottom sharing similar habitat preferences. The Bhattacharya’s method [34] was used to separate the first modal component of the 0 age group from the annual standardized trawl survey length frequency distributions (LFDs). The 0 group threshold limit was defined using growth data available in the Mediterranean and routinely collected within the EU data collection framework (DCF). In some circumstances, when the splitting of modal components in the LFDs was difficult (e.g. low number of recruits), recruits were computed using a fixed threshold length derived from validated local studies. A summary of the data source, methods applied and threshold lengths used for recruits identification is provided in S1 Table.

The only exception to this framework of recruit identification was for common sole in GSA 17. For this population, the SoleMon survey sampled the 0 group juveniles when they migrate offshore from estuaries and coastal lagoons that are considered the core nursery habitats of the population [32]. Due to lack of data on small-sized sole in nursery areas, we addressed the position of open water juvenile habitats, where the largest specimens of the 0 group concentrated at the end of their inshore life. This information was considered crucial from a fishery management perspective to provide guidance for the protection of relevant juvenile habitats exposed to trawling activities.

As a second step in our framework we used the identified threshold length of recruits of each target species to calculate the density indices (n km^-2^) of recruits in each sampling station as a base to model their annual spatial distribution.

### Spatial modelling

From the results of the preliminary modelling trials carried out on a subset of species in several areas, it was clearly recognized that identification of a unique modelling approach/methodology suitable for all species/stages/areas was unrealistic. The amount of data (i.e. positive hauls), environmental characteristics of the areas and the availability of appropriate covariates were all factors that varied significantly across species and areas, in turn reducing the possibility to standardize the applied methods consistently. Spatial distribution modelling was also constrained by the number of positive hauls (i.e. hauls with occurrence of recruits of a given species) occurring for the different species and areas and proved unfeasible in many cases.

In our analytical framework we adopted the generalized linear model (GLM, [35]) as the main method for modelling the distribution of recruits. Model selection was performed with a forward stepwise selection procedure. Starting from the null model, each covariate was added individually; the predictor resulting in the highest AIC (Akaike Information Criterion) decrease or, depending on the model type, the better cross-validation index (CVI, S1 Text), was included in the model. The latter model was chosen to start the next step where the procedure was repeated until it was no longer possible to reduce AIC or improve CVI by including other available covariates. These included latitude, longitude, sampling depth, bottom steepness and distance from the coast. The latter two were obtained from the Marspec dataset [36].

When a spatial structure was evident in GLM residuals, different spatial correlation structures (e.g. exponential, spherical) were successively considered and the structure that best fitted the observations was identified [37]. This correlation structure was then incorporated as a random effect of a generalized linear mixed model (GLMM, [38]) where the final predictions were made by adding the predicted deterministic part of variation and the interpolated residual variation. Ordinary kriging (OK [37]), eventually replaced by the inverse distance weighting (IDW [39]) in case of poor fit of the variogram, was used to interpolate model residuals.

When the percent of total variance explained by a GLM/GLMM model was not satisfactory, we moved to other classes of models, first generalized additive models (GAM [40]) and then zero-inflated generalized additive models (ZIGAM [41]). In both cases we followed the same approach used for GLM to account for spatial autocorrelation in model residuals.

Finally, geostatistical methods, i.e. OK, and eventually IDW in case of poor variogram fit, were used when any of the regression models applied returned reliable spatial predictions.

Table 1 summarizes the modelling performed for recruits of the 11 selected species in the different Mediterranean sub-areas.

### Generalized linear models (GLM) and generalized linear mixed models (GLMM)

GLM assumes that the dependent variable follows a distribution within the exponential family (e.g. normal, exponential, binomial) and a function of the mean (link function) depending linearly on some covariates.

The dependent variable in our case was the log of the density on site *v* and time t (*y(v*,*t)*) plus 1. The model used is:

$$E\left(\log\left(y\left(v,t\right)+1\right)\right)=\beta_{0}+\overset{p}{\underset{j=1}{\sum }}\beta_{j}x_{j}\left(v,t\right)$$

where the *x*_*j*_ *(v*,*t)*s are the covariates, *β*_*0*_ is the intercept, *β*_*j*_ are the regression coefficients of the covariates.

When the variogram fit of the residuals showed spatial auto-correlation this was taken into account adopting a GLMM [38] where the final predictions were made by adding the predicted deterministic part of variation and the interpolated residual variation. Residuals were interpolated using ordinary kriging, eventually replaced by IDW, with the power coefficient generally fixed as 5, in case of poor variogram fit.

The general computation of this model is:

$$E\left(\log\left(y\left(v,t\right)+1\right)\right)=\beta_{0}+\overset{p}{\underset{j=1}{\sum }}\beta_{j}x_{j}\left(v,t\right)+\text{W}\left(v,t\right)$$

where W(*v*,*t*) is the random effect that is assumed to be normally distributed.

In two specific case, recruits of the blackmouth catshark (*G*. *melastomus*) and deep-water rose shrimp (*P*. *longirostris*) in the Ligurian and North Tyrrhenian Seas (GSA 9), modelling issues raised by the high number of 0 observations were addressed by implementing a Bayesian GLMM with the INLA (Integrated Nested Laplace Approximations) approach [42]. The prior distributions of the parameters of the random effect were defined using an estimation chain where the posterior distributions of one year were used to define the prior distributions of the following year as in [25].

### Generalized additive models (GAM) and generalized additive mixed models (GAMM)

The GAM is an extension of GLM, in which the linear form of the covariates function is replaced by a smooth function. The smooth functions used are generally cubic splines.

As for GLM, the dependent variable is *log*(*y*(*v*,*t*)+1) and the model was:

$$E\left(log\left(y\left(v,t\right)+1\right)\right)=\beta_{0}+\overset{p}{\underset{j=1}{\sum }}s\left(x_{j}\left(v,t\right)\right)$$

where *s*(*x*_*j*_(*v*,*t*)) is a non linear effect on the covariate.

Also in this case we checked for spatial autocorrelation in the residuals and, if needed, a kriging was accomplished to interpolate residuals variation to be incorporated into the model. In the latter case we have a GAMM with the following formulation:

$$E\left(log\left(y\left(v,t\right)+1\right)\right)=\beta_{0}+\overset{p}{\underset{j=1}{\sum }}s\left(x_{j}\left(v,t\right)\right)+\text{W}\left(v,t\right)$$

where, as in GLMM, W(*v*,*t*) is the random effect.

### Zero inflated generalized additive models (ZIGAM)

One of the main problems in the analysis was dealing with the large amount of zero catches. ZIGAMs assume that the response variable follows a probabilistic mixture distribution of a zero atom and a continuous distribution belonging to the exponential family [41].

The data have been modelled in two steps. First the probability (*P*) of observing a density greater than 0 is modelled with a GAM where the dependent variable has a binomial distribution and the link function is the logit (logarithmic of the probability of observing a positive catch on the probability of observing a zero):

$$logit\left(p\left(v,t\right)\right)=\beta_{0}+\overset{p}{\underset{j=1}{\sum }}s\left(x_{j}\left(v,t\right)\right)$$

Then a GAM model is estimated only on the positive catches using a Gaussian family model, the identity link function and *log*(*y*(*v*,*t*)) as dependent variable.

$$E\left(log\left(y\left(v,t\right)\right)\right)=\beta_{0}+\overset{p}{\underset{j=1}{\sum }}s\left(x_{j}\left(v,t\right)\right)$$

As in GLM and GAM we checked for spatial autocorrelation in the residuals and, if needed, an OK or IDW was used to interpolate residual variation to be incorporated into the model as random effects.

### Kriging

Ordinary kriging was used to model the recruits of red mullet (GSA 18: Southern Adriatic Sea), common sole (GSA 17), common Pandora (GSA 10: South Tyrrhenian Sea; GSA 11: Sardinia, GSA 18), Norway lobster (GSA 11, GSA 19: Western Ionian Sea) and broadtail shortfin squid (GSA 17, Table 1).

The OK model structure can be formalised as:

log(y(v,t)+1)=μ(v)+W(v,t)

where μ(*v*) is the spatial trend (or large-scale variation).

### Identification of annual nurseries and persistence analysis

As a third step of our approach to locate and classify nurseries, we identified the annual density hot-spots of recruits, which were regarded as annual nursery areas. The Getis’ G statistic [43], with a radius of 2.5–5.0 km and a 0.95 level of significance, was selected among the local methods for spatial hot-spot identification. This approach was applied separately in each GSA and for each year of the time series to spatially locate clusters of recruits displaying a significant higher density.

Finally, the Index of Persistence (I_i_) measuring the relative persistence of the cell *i* of a given GSA as an annual nursery [25, 44] was calculated in each 1 *km* ^2^ cell considering *δ*_ij_ = 1 if the grid cell *i* was included in a nursery in the annual survey *j*, and *δ*_ij_ = 0otherwise:

$$I_{i}=\frac{1}{n}\overset{n}{\underset{\text{j}=1}{\sum }}\delta_{ij}$$

where *n* is the number of annual surveys considered in that GSA. The index I_i_ ranges between 0 (cell *i* never included in an annual nursery area) and 1 (cell *i* always included in an annual nursery area) for each cell of the GSA. Results were plotted in the persistence maps reporting five classes (0.05–0.20, 0.21–0.40, 0.41–0.60, 0.61–0.80 and 0.81–1). These maps allowed us to compare the nurseries persistence of the 11 target species at the scale of EU Mediterranean waters independently by the predicted recruit abundance.

### Multispecies analysis of nursery area overlap

The spatial overlap between the identified nursery areas at different levels of temporal persistence (*I*_*i*_ >0.05, *I*_*i*_ >0.2; *I*_*i*_ >0.4) was examined to identify key recruitment areas in a multi-species context. In particular, the following levels were considered. In each GSA, every cell *i* was classified according to its importance for the recruitment of the set of species, considered using the following index of spatial overlap between recruitment areas:

$$SO_{i}=\frac{1}{k}\overset{k}{\underset{\text{j}=1}{\sum }}\delta \left(I_{ij}\right)$$

where *k* is the total number of modelled species showing *I*_*ij*_ > n^th^-level in the GSA and *δ*(*I*_ij_) = 1 if *I*_ij_ > n^th^ level in the cell *i* for the species *j*, and 0 otherwise. The index was applied in those GSAs where more than two species were modelled and classified each cell of a GSA between 0, no spatial overlap of persistent (*I*_*i*_ > n^th^-level) nurseries and 1, complete spatial overlap.

### Conservation effect of the current fisheries restricted areas (FRAs)

**Fig 2:**
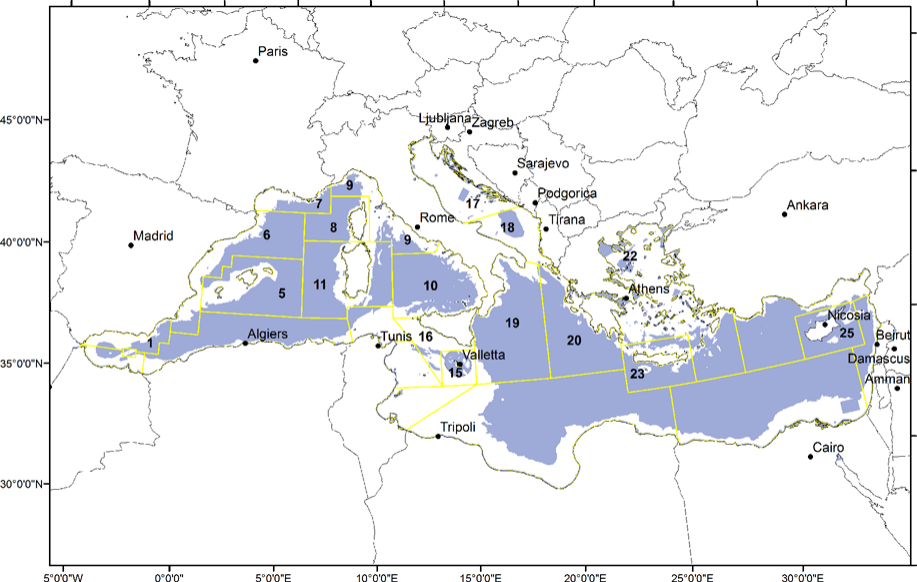
Trawl fishery restricted areas in the Mediterranean Sea. Trawl fishery restricted areas are shown in blue, trawlable areas are shown in white.

To evaluate the effect of the on-going temporal or permanent restrictions of trawl fishing activities in EU Mediterranean waters, we incorporated outcomes from the MEDISEH project. In particular, we used the data gathered from more than 200 trawl fishery restricted areas (TFRA) across the EU Mediterranean basins, where some sort of spatial (12 months per year) or temporal (>2 months per year) prohibition is applied (Fig. 2). TFRAs included trawl fishery restricted areas that may also have designations as marine protected areas under national legislation or have an international designation as TFRA by the EU/GFCM.

The spatial overlap between nurseries and TFRAs was calculated for each species to obtain the proportion of nursery areas currently under some sort of protection from trawling.

## Results

The number of species for which it was possible to identify nurseries varied among GSAs ranging between 2 (Balearic Islands: GSA 5) and 9 (Sardinia: GSA 11). For some species (e.g. red mullet, common Pandora), the modelling of recruit density was generally unfeasible, due to the lack of temporal overlap between the survey and recruitment periods of target species in the different Mediterranean areas, with the exception of the Adriatic Sea (GSA 17 and 18) where the MEDITS surveys generally occurred later than in other GSAs. In other cases, the abundance during MEDITS surveys was scanty (e.g. thornback ray) or recruitment areas were not covered by the survey (e.g. giant red shrimp in some areas). Finally, in Corsica (GSA 8) and Cyprus (GSA 25) the MEDITS hauls were few, scattered and generally characterized by catches of juveniles that were too scanty to be used for modelling. The final models that were chosen and their goodness-of-fit are listed in S2 Table.

### European hake

**Fig 3:**
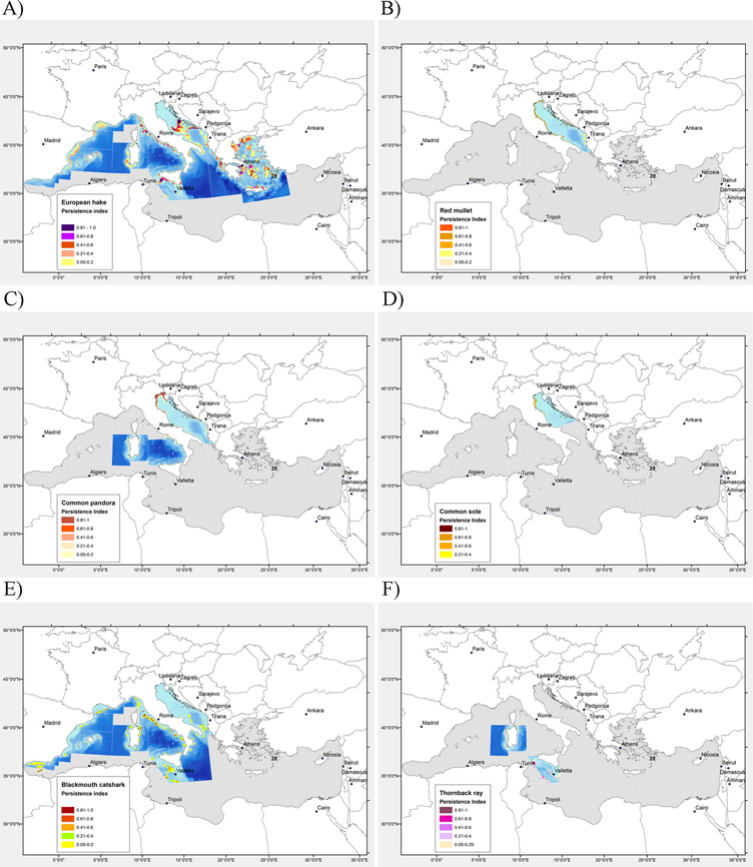
Distribution maps of persistence index (Ii) of nursery areas of commercial fish in the North Mediterranean Sea. A) European hake (*Merluccius merluccius*), B) red mullet (*Mullus barbatus*), C) common Pandora (*Pagellus erythrinus)*, D) common sole (*Solea solea*), e) blackmouth catshark (*Galeus melastomus*) and E) thornback ray (*Raja clavata*). The index *Ii* ranges between 0 (portion *i* of the study area never included in an annual nursery) and 1 (portion *i* of the study area always included in an annual nursery).

The threshold size of hake recruits ranged between 8.5 and 14.5 cm total length (TL). Nursery areas were mostly found between 100 and 250 m depth, with a patchy distribution along the shelf break (Fig. 3a).

Along the Spanish coast the largest nurseries were identified between the Ebro River delta and Cape Nao between 150 and 250 m depth. Around the Balearic Islands, recruits were found at a low density north-west of the Menorca channel, and south-west of Mallorca. In the Gulf of Lions (GSA 7) recruits were found at the external border of the continental shelf, in the vicinity of the heads of canyons where a Fishery Restricted Area was established in 2008. A series of persistent relatively small but high density patches were found along the western coast of Italy (GSA 9: Ligurian and North Tyrrhenian Seas and GSA 10), on both sides of the Southern Adriatic Sea and Western Ionian Sea. Similarly, several areas of the Aegean Sea—Crete Island (GSA 22–23) and Eastern Ionian Sea (GSA 20) were characterized by high density areas of recruits with intermediate (*I*_*i*_ = 0.41–0.6) or high (*I*_*i*_>0.6) temporal persistence, the most relevant being the one found in Saronikos Gulf. A less fragmented spatial pattern of nursery distribution was found in the Strait of Sicily (GSA 15–16), where the main nursery area was located to the east of the Adventure Bank extending also along the central-southern coast of Sicily, and off the south-western Sardinian coast (GSA 11).

Large persistent high density patches were also found in the central-northern Adriatic Sea (GSA 17) where three main persistent nursery areas were found respectively southwards and eastwards of the Middle Adriatic Pit and around the north-eastern margin of the South Adriatic Pit.

### Red mullet

The timing of the MEDITS survey did not match the recruitment period of *M*. *barbatus*, as juveniles appeared mostly during late summer. However, in the Adriatic Sea (GSAs 17 and 18) it was possible to observe significant aggregation of recruits and consequently model their distribution (Fig. 3b). The threshold size of recruits in such areas ranged between 6.0 and 8.8 cm TL. In the Northern Adriatic a persistent recruitment area was identified between 10 and 20 m depth over the sandy-mud coastal areas of the Italian side. In the Southern Adriatic a main hot-spot of recruits was found offshore of the Gargano promontory, while a smaller one was observed in front of Bari (Apulia region).

### Common Pandora

Persistent hot-spots of recruits of common Pandora were only successfully modelled along the coastal sandy areas of GSAs 17, 18, 10 and 11 (Fig. 3c). The threshold size of recruits averaged between 6.0 and 14.6 cm. Nursery areas were only evident in the central/northern Adriatic (GSA 17), with a large nursery spreading from the north-eastern Italian coast to the eastern coast of Istria (Croatia) at a depth of 5–20 m. Another important area was identified along the Southern Croatian coast. In the Southern Adriatic, South Tyrrhenian Sea and Sardinia, nursery areas generally appeared to be small and poorly persistent with a few exceptions (e.g. a small patch along the N Albanian coast).

### Common sole

Common sole habitats could only be studied in GSA 17, where a time series of beam trawl survey data (SoleMon survey) was available. The threshold size of sole juveniles ranged between 17.7 and 20.4 cm TL. Their core distribution was located around the mouth of the Po River, whilst the southward extension of that area varied between years (Fig. 3d).

### Blackmouth catshark

Nursery areas of *G*. *melastomus* were identified in all GSA areas, except in the waters surrounding Corsica (GSA 8), Cyprus, the Northern Adriatic, where the species is not present, and the Greek GSAs (GSAs 20–22–23, Fig. 3e). The assessment of threshold sizes for recruit identification varied among the areas, from a minimum value of 13.5 cm calculated in Greek waters, to a maximum of 28.5 cm TL applied in the Strait of Sicily. Persistent recruitment areas appeared as a long and almost continuous strip along the upper slope (200–600 m) off the western coast of Italy (GSAs 9 and 10), Gulf of Lions and the north-western coast of Sardinia (GSA 11). Along the Iberian Peninsula coastline (GSAs 1 and 6), most nursery areas showed a low temporal persistence except for one nursery located in the middle of the Alboran Sea and on the upper slope off the Balearic Islands, mainly to the north of Menorca Island and south-west of Mallorca.

Only scattered and poorly persistent nursery grounds were found in the north-western Ionian Sea, whereas on the upper slope of the neighbouring GSA 18, an area with a medium persistence (*I*_*i*_ = 0.4–0.6) appeared along the border of the South Adriatic Pit.

Three nursery areas with medium persistence were found on the upper slope of GSAs 15 and 16. The two largest ones were located to the north and south of the Malta Graben, and a smaller one was located to the south of Malta.

### Thornback ray

Due to infrequent catches of thornback ray recruits, even in areas with rather high densities of adults (i.e. Corsica, Balearic Islands; [45]) modeling of the density data was feasible in only three GSAs (11, and 15–16). The threshold size of recruits ranged from 35–41 cm TL. Very few persistent nursery grounds were identified, with the more important areas located in the Strait of Sicily (GSAs 15–16) at depths up to 400 m (Fig. 3f). The most stable density patch occurred offshore from the south-western coast of Sicily, in an area of intense hydrographic activity due to the eastward surface flow of Modified Atlantic Water and the westward flow of Levantine Intermediate Water. High density patches of recruits were also found around Sardinia, at depths of 100–300 m (Fig. 3f).

### Giant red shrimp

**Fig 4:**
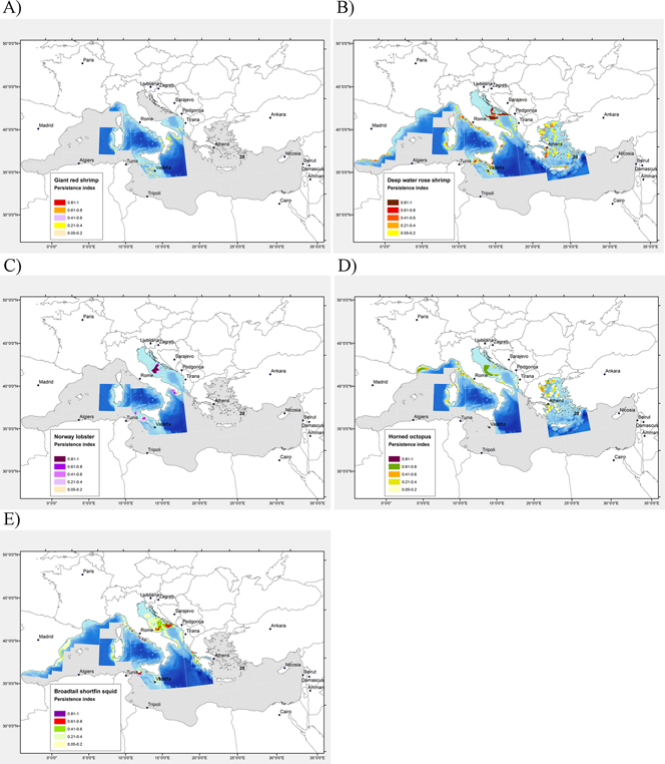
Distribution maps of persistence index of nursery areas of commercial species of crustaceans and cephalopods in the North Mediterranean Sea. A) Giant red shrimp (*Aristeomorpha foliacea*), B) deep-water rose shrimp (*Parapenaeus longirostris*), C) Norway lobster (*Nephrops norvegicus)*, D) horned octopus (*Eledone cirrhosa*), E) broadtail shortfin squid (*Illex coindetii*). The index *Ii* ranges between 0 (portion *i* of the study area never included in an annual nursery) and 1 (portion *i* of the study area always included in an annual nursery).

Giant red shrimp nurseries were identified in the central Mediterranean Sea, including South Tyrrhenian Sea, Sardinia, Strait of Sicily, Southern Adriatic Sea and Western Ionian Sea, Fig. 4a. MEDITS catches from the Western Mediterranean (GSAs 1, 5–7), the Northern Adriatic and Cyprus were extremely low. Around Corsica Island, in the Ligurian and North Tyrrhenian Sea, as well as in the Eastern Ionian, Aegean Sea and Crete Island, giant red shrimp where somewhat more abundant, but nevertheless the catch of recruits was insufficient for nursery grounds identification.

The average threshold size of recruits in the areas varied between 25 mm and 31 mm carapace length (CL). The depth range of the most persistent density hot-spots generally varied between 500 m and 700 m depth, although a few nurseries were found to be located in shallower waters on the upper continental slope starting at depths of 250 m.

In the South Tyrrhenian Sea (GSA 10) nursery areas with an intermediate level of temporal persistence (*I*_*i*_ = 0.4–0.6) were localized off the Calabrian coast. Around Sardinia, nursery areas were found both on the east and west side, with the most persistent patch of recruits offshore of the south coast. In the Strait of Sicily (GSA 15–16), recruits were abundant in a wide area with an intermediate to high level of persistence (*I*_*i*_ = 0.4–0.8) off the south to south-west coast of Malta. Two persistent patches of recruits were found south of the Otranto channel (GSA 19) and off the southern border of the Adriatic Pit (GSA 18).

### Deep-water rose shrimp

Most GSAs in the Mediterranean were characterized by high abundances of recruits of *P*. *longirostris*. Exceptions were the Balearic Islands, the Gulf of Lions, Corsica, the Eastern Ionian and Cyprus, where the few positive hauls in each annual survey and/or the low amount of individuals captured did not allow the distribution of nursery areas to be modelled. Overall, mean length of recruits ranged between 10 and 20 mm CL. Nursery areas were mostly located on muddy bottoms along the shelf break-upper slope between 100–200 m and even deeper in some areas (Fig. 4b). A single wide nursery area occurred all along the South Spanish coast (mainly in GSA 1), with the highest persistence (*I*_*i*_ = 0.6–0.8) found in the eastern North Alboran Sea. Off the Italian coast nurseries appeared as highly persistent (*I*_*i*_ = 0.8–1.0) patches, in particular in the Tyrrhenian Sea (GSA 9 and 10), western coast of Sardinia, Strait of Sicily and Western Ionian Sea. Three large areas of high persistence (*I*_*i*_ = 0.8–1.0) were identified for the recruits of deep-water rose shrimp in the Northern Adriatic. They extend south east of the Middle Adriatic Pit until the southern limit of GSA 17. In the Aegean Sea and Crete Island the most persistent density hot-spots (*I*_*i*_ = 0.4–0.6) were identified in offshore deep areas of Argolikos Gulf (west-central Aegean), in the Thracian Sea (northern GSA 22) and on the northern Cretan shelf (GSA 23).

### Norway lobster

Norway lobster recruits were below 20–30 mm CL, depending on the area. Nursery areas were found at depths ranging from 100 to 600 m on the north-eastern coast of Sardinia, in the Strait of Sicily, the Northern Adriatic Sea and along the south-eastern coast of Italy (north-western Ionian Sea, Fig. 4c). Very low MEDITS catches of juveniles were obtained in the remaining GSAs. Along the upper slope of Sardinia nursery areas were identified as thin and relatively poorly persistent patches at depths ranging from 400 to 600 m. In the Strait of Sicily recruits were distributed on muddy bottoms of the upper slope between 250 and 500 m. Two major large offshore nurseries were identified along the south-eastern coast of Sicily. Another large and persistent (*I*_*i*_ = 0.4–0.6) offshore recruitment area was found in the Western Ionian Sea on the Apulian coast (Italy).

In the Northern Adriatic Sea a wide area where small-specimens concentrate was identified in correspondence with the Middle Adriatic Pit at depths ranging from 100 m to 270 m.

### Horned octopus

Recruits were identified using a threshold size between 30 and 56 mm mantle length (ML). Along the Spanish coast and the Strait of Sicily MEDITS catches of juveniles were too low to develop reliable spatial modelling. Modelling showed the occurrence of several small and persistent density hot-spot areas along the shelf-break off the north-western coast of Italy (GSA 9) and in Sardinian waters, mainly over detritic bottoms colonized by crinoid beds between 100 and 180 m depth (Fig. 4d). In the central-southern Tyrrhenian Sea (GSA 10) two very persistent (up to 100%) hot-spots were localised in the Gulf of Gaeta and Napoli.

Nurseries appeared as large and highly persistent patches from the shelf-break to upper slope of the Gulf of Lions and in the Northern Adriatic. Here a wide offshore recruitment area was identified between 60–160 m depth on the south-western side of the GSA. This area appeared connected with a series of small patches of recruits on the margin of the Adriatic Pit (GSA 18). A second, smaller area was found westwards of the Croatian Islands at a depth of 120–150 m (Fig. 4f). In the Greek Seas (GSAs 20, 22–23) the main nurseries were found in the north-western part of the Aegean Sea, mainly on the extended continental shelf of Thermaikos Gulf (Fig. 4d).

### Broadtail shortfin squid

Nurseries of the broadtail shortfin squid were identified in most of the GSAs using a threshold size of recruits between 61 and 115 mm ML. Along the Spanish coast recruits were more abundant in the Catalan Sea (GSA 06), with the highest abundance observed between 100 and 200 m depth. In the Ligurian and North Tyrrhenian Sea recruits were distributed in several small patches along the shelf break mainly between 70 and 150 m depth (Fig. 4e). Two main, rather persistent (*I*_*i*_ = 0.4–0.6), nursery areas appeared offshore in the central and southern sectors of the western Sardinian coast, between depths of 100 and 300 m, with an increased abundance over the detritic bottoms at the shelf-break. Similarly, in GSA 10, persistent nursery areas (*I*_*i*_ = 0.4–0.6) were localized on detritic bottoms in the Gulf of Gaeta and off Capo Bonifati between 70 and 150 m depth (Fig. 4e). In the Strait of Sicily, the main nursery areas were located offshore off the south-east edge of the Adventure Bank (South-East Sicily). Another important nursery occurred along the eastern edge of the Malta Bank (Fig. 4e). In the Northern Adriatic there was a wide recruitment area extending between 50 and 300 m depth with two more persistent patches. Nurseries in the Southern Adriatic Sea (GSA 18) mainly occurred along the Italian side, between 100 and 150 m depth. Small and persistent high density patches of recruits were also identified in the Ionian Sea both on the Italian (GSA 19) and Greek (GSA 20) sides, with the most persistent areas being outside Thermaikos Gulf in the Northern Aegean (GSA 22).

### Multispecific spatial overlap between nursery areas

**Fig 5:**
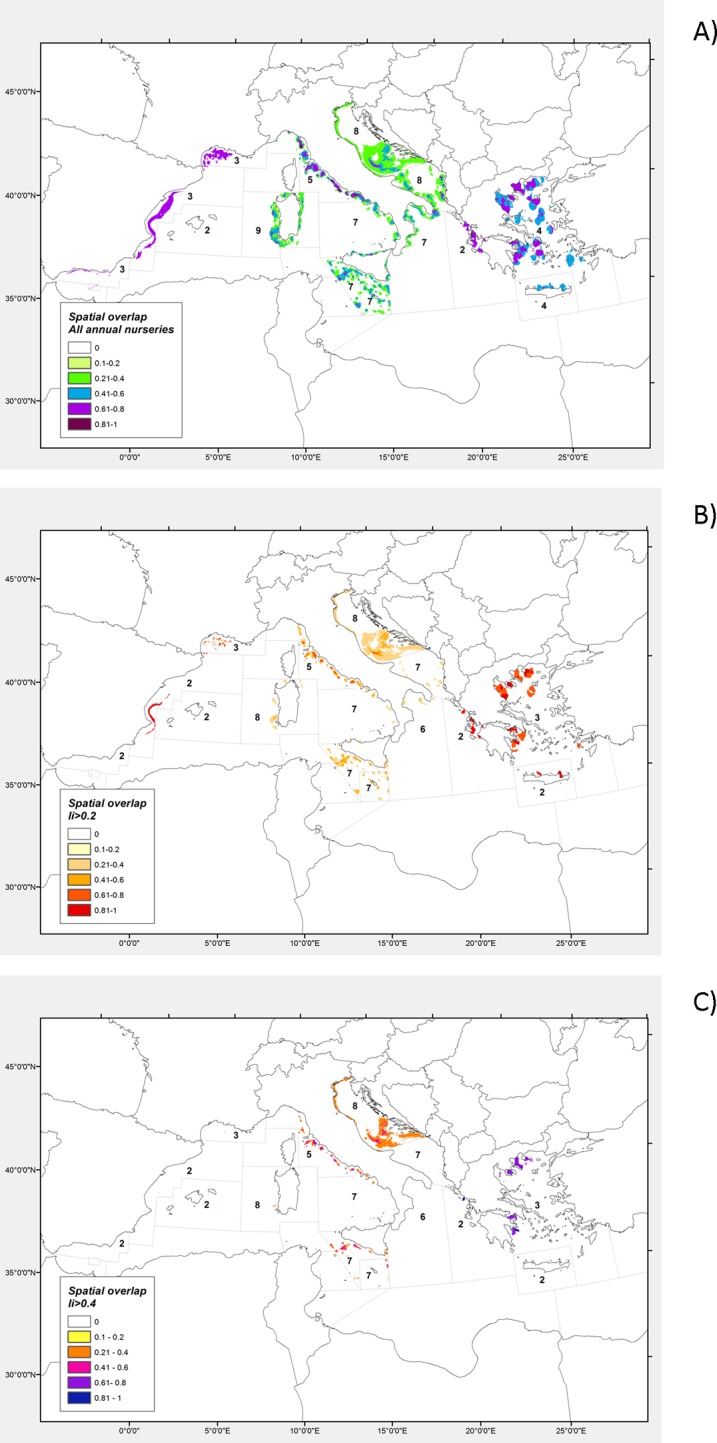
Map showing the spatial overlap among the persistent nursery areas of 11 species of demersal fish and shellfish in EU Mediterranean waters. Colours indicate different classes of spatial overlap index (*SO*). Spatial overlap was calculated considering three different classes of the temporal persistence index (*I*_*i*_): A) *I*_*i*_>0.05; B) *I*_*i*_>0.2; C) *I*_*i*_>0.4. Numbers indicate the numbers of species modelled in each GSA. Note that, since the number of species modelled varied among GSAs, the overlap index *SO* was calculated separately for each GSA to avoid any comparative analysis at a larger geographical scale that can lead to misleading conclusions.

The analysis of the spatial overlap of the nurseries identified at different persistence levels (*I*_*i*_ >0.05, *I*_*i*_ >0.2 and *I*_*i*_ >0.4) allowed the identification of several areas across the Mediterranean that provided multispecies critical habitats, and hence the exploration of different scenarios (Fig. 5). No overlap analysis was performed in GSA 5 where only two species were modelled.

It is important to note that, since the number of species modelled varied among GSAs, the overlap index *SO*_*i*_ can be analysed only at the GSA level, avoiding any comparative analysis of its values at a larger geographical scale that can lead to misleading conclusions. However, the emergence of some recurrent, and even important, patterns of overlap can be highlighted.

As expected, the overlap areas decreased drastically when increasing the level of persistence from *I*_*i*_ >0.05 (Fig. 5a) to *I*_*i*_ >0.4 (Fig. 5c). Several patches of co-occurrence of nursery areas of multiple species were identified for *I*_*i*_ >0.4 only along the coast of the Ligurian and North Tyrrhenian Sea, Northern Adriatic Sea, Strait of Sicily, Aegean Sea and Crete Island (Fig. 5c). The lower interspecific spatial overlap observed in the other GSAs can be attributed to both a low level of persistence of many of the species and a considerable spatial mismatch among the most persistent nursery areas of the different species.

Nursery areas of European hake, deep-water rose shrimp, broadtail shortfin squid and horned octopus co-occurred along the shelf-break with spatial difference in temporal stability, from *I*_*i*_ >0.2 off the south-west Sardinia and the northern sector of the south Tyrrhenian (Fig. 5b) to *I*_*i*_ >0.4 in the central-northern Tyrrhenian Sea and central Adriatic (Fig. 5c). In the other GSAs, the nursery areas of these 4 species, when identified, also showed a clear spatial association. The overlap area was due to the co-occurrence of European hake and broadtail shortfin squid along the Spanish coast and in the Eastern Ionian for *Ii* >0.2, also associated with the deep-water rose shrimp in the Strait of Sicily and along the South East Sicilian coast (GSA 19) and with the blackmouth catshark in the Gulf of Lions and in the northern sector of GSA 10. The nurseries of European hake were found in partial overlap with the nurseries of the two cephalopods in the North-West side of GSA 18 while on the east side of the GSA, along the Albanian coast, the species prevalently co-occurred with the deep-water rose shrimp. Varying degrees of overlap between nurseries areas of European hake and deep-water rose shrimp in association with the horned octopus were also found in the Aegean Sea and Crete and in North Sicily (GSA 10). A degree of overlap between the distribution of broadtail shortfin squid and deep-water rose shrimp recruits was observed in the Northern Alboran Sea (GSA 1) independently of persistence level (*Ii* >0.05, Fig. 5a). Overlap of stable nurseries of the more coastal species, red mullet, common Pandora and common sole were exclusively observed in the Italian waters of the Northern Adriatic. Finally, with regard to bathyal species, patches of co-occurrence of giant red shrimp and Norway lobster were identified in the Western Ionian and in the deep basin between the two banks of the Strait of Sicily, sometimes in association with the blackmouth catshark. A nursery area of blackmouth catshark partially overlapped a nursery area of giant red shrimp on the deep grounds of the northern sector of GSA 10.

### Proportion of nurseries under fisheries restrictions

The on-going spatial measures of fisheries restrictions for trawl fisheries (TFRA) in the Mediterranean Sea showed a significant conservation effect mostly for species with nurseries on coastal grounds shallower than 50 m. This was the case of red mullet, common Pandora and common sole with 66.8%, 54.1% and 46.1% of persistent nursery areas under protection (Table 2). On the other hand the proportion of nursery areas protected was much lower for European hake (15.3%) and Norway lobster (17.8%), whose recruitment occurs on the shelf-break and upper slope below 150 m. Finally, there was minimal overlap between TFRA and recruitment areas for offshore species (i.e. thornback ray, blackmouth catshark, giant red shrimp and deep-water rose shrimp) distributed over the continental slope deeper than 200 m.

**Table 2:** Surface extent in km2 of total nursery areas identified and of nursery areas with a temporal persistence >40% (N40), for a set of 11 demersal species in Mediterranean European waters.

*Species*	Overall nursery habitat area (km^2^)	Nursery habitat area (N40, km^2^)	Total overlapping area (km^2^) between TFRA and N40	Percentage of N40 protected by TFRA
*M*. *merluccius*	135273	31518	4822	15.3
*M*. *barbatus*	11074	6712	4482	66.8
*P*. *erythrinus*	18738	6803	3683	54.1
*R*. *clavata*	15950	1783	30	1.7
*G*. *melastomus*	51588	7727	282	3.7
*S*. *solea*	2894	2048	944	46.1
*A*. *foliacea*	27288	1687	0.1	0.0
*P*. *longirostris*	90190	22474	1286	5.7
*N*. *norvegicus*	27613	10861	1936	17.8
*E*. *cirrhosa*	89602	25727	2999	11.7
*I*. *coindetii*	116760	36869	2390	6.5

The conservation effect of ongoing spatial restriction measures for trawling (TFRA) is measured in terms of total and percentage of protected N40.

## Discussion

Our study provides the first comprehensive view of the distribution and temporal persistence of nurseries of 11 important demersal species in the European Mediterranean Sea, based on 17 years of annual bottom trawl surveys. The most important outcome of the analysis is the assessment of the relevance of different areas of the Mediterranean as recruitment areas both for single and multiple species, thus forming the knowledge-base for the development of a spatial planning approach to the management of fishing activities.

This is a novel aspect of our study considering that very few studies have spatially addressed nursery habitats simultaneously for multiple species. In an integrated assessment of the differential fish nursery functions of the main estuaries along the Portuguese coast, Vasconcelos et al. [46] suggested that management and conservation of estuaries should focus on sites with higher contributions to adult sub-populations of multiple species.

In our modelling framework, GSAs were adopted as spatial units for the analyses considering that the ongoing regional fisheries management approach developed by the GFCM, the FAO regional body responsible for the implementation of management measures in the Mediterranean and Black Sea, is based on using GSAs as management units.

For the purpose of identifying nursery areas, the presence of high concentrations of recruits and their temporal stability were considered as basic justification of the methodological approach [25], even though the effective contribution of persistent nursery habitats to adult populations needs to be further addressed.

A great effort was made to standardize the protocol of analysis, although differences emerged among GSAs in the number of species for which it was possible to identify nurseries, as well as in the spatial modelling approach adopted to map recruits density. This was based on a conceptual and adaptive framework of model selection that includes both regression approaches (e.g. GLM, GAM) and standard geostatistical techniques (e.g. kriging). The use of a unique standardized method of spatial modelling was unfeasible, mostly due to the large observed differences in the availability of data (e.g. occurrence and abundance of recruits) and environmental predictors for the different combinations of species and areas. Our model framework flexibility has reduced the possibility to compare density estimates between areas without substantially impairing the spatial identification of key nursery areas in each GSA. As an add-on to this novel combined assessment, the local experts included in this study, evaluated model results and assembled these results into a North Mediterranean-wide assessment through extensive discussion and knowledge-result sharing.

The species included in the analyses ranged from a minimum of two in the Balearic Islands (GSA 5) to a maximum of nine species in waters surrounding Sardinia (GSA 11). The main reasons were either the temporal mismatch between bottom trawl surveys and recruitment periods of the selected species (e.g. red mullet) or the low abundance of recruits (e.g. thornback ray).

Variability in the observed recruits’ abundance among GSAs was due to factors such as geographical distribution patterns, occurrence of appropriate habitats and, possibly, effects of fishing pressure. The latitudinal and longitudinal change in abundance across the Mediterranean explains, for instance, the absence of identified nurseries for the deep-water rose shrimp and giant red shrimp. These two commercial crustaceans display an increase in abundance along a north-south gradient and, in the case of the giant red shrimp also an almost total absence from north-western areas [47–49]. The effect of fishing pressure, combined with a lack of suitable habitats can explain the observed low abundance of recruits of species such as the thornback ray and common sole in most of the areas. The former species, as with the majority of elasmobranchs, has declined in many areas during the last 40 years, only remaining abundant in a few Mediterranean sectors, such as the Strait of Sicily and Sardinia, where defined nurseries can be still identified [50–51]. In the case of common sole, survey data allowed the identification of nursery areas only in the Adriatic Sea where one of the main Mediterranean stocks can be found [52].

It is however important to note that the identification of nurseries was based on the MEDITS time series (1994–2010). This implies that we have identified areas of aggregation of recruits and measured their temporal stability during late spring-early summer, i.e. the period of the year when the MEDITS survey is carried out. This is an important recruitment season for many Mediterranean species whose spawning peak is mostly in spring (see [53] for a review on this subject).

On the other hand, there was a mismatch between the MEDITS survey period and the recruitment peak for some species spawning in late spring-summer, such as red mullet and common Pandora [53], whose nurseries were only identified in the Adriatic Sea. The MEDITS catch of recruits of these two species in the Northern Adriatic Sea was probably due to a delay in the survey period to late July-August that occurred in some years. Further investigation would therefore be necessary to elucidate the dynamics of recruitment across seasons, in particular for those species with a long spawning period, such as hake or the deep-water rose shrimp. Previous studies on hake nurseries in the Western Mediterranean basin, however, have shown a general spatial stability of the main nurseries during the year [21–25].

Given the wide bathymetric range of the studied species, the areas identified as nurseries encompassed different habitats, from sandy coastal shores, where recruits of red mullet and common Pandora aggregate, to the upper slope muddy bottoms hosting the nurseries of slope species such as Norway lobster, giant red shrimp and blackmouth catshark. However, a high degree of overlap was generally observed among nurseries of species with similar depth preferences. We found a high spatial overlap between the persistent nurseries of some of the species occurring on the shelf-break (120–170 m), indicating the predominant effect of local circulation patterns for larval transport and retention for species such as hake, deep-water rose shrimp, broadtail shortfin squid and horned octopus [54]. In the central Mediterranean, the importance of detritic bottoms on the shelf break for the recruitment of several demersal species has already been pointed out [11]. Shelf-break upwelling [55] and water turbulence drive both an increase in organic matter transportation and nutrient input into the water column, which in turn lead to an increased abundance of epibenthic filter feeders, in particular the crinoid *Leptometra phalangium*, and nektobenthic organisms feeding upon zooplankton and epibenthos [21, 56–59]. In this study we showed that the shelf break plays a role as a nursery area in several Mediterranean sectors such as the eastern border of the Adventure and Malta Banks in the Strait of Sicily, the South Ligurian and the Tyrrhenian seas, the South-Western Sardinian coast, and the south-eastern sector of the Gulf of Lions where hake recruits appeared along the outer border of the continental shelf, close to the heads of canyons where a Fishery Restricted Area has been established in 2008 [60]. Furthermore, a key recruitment area occurs along the shelf break region (150–250 m) offshore of the North-Western Spanish coast (GSA 1–6) between the Ebro River delta and Cape Nao. This is an area with a wide continental shelf, characterised by frequent upwelling events [61] and important land runoff, where temporary discharges due to storms may produce intense local enrichment [62]. The effect of local enrichment due to fresh water inflow from the Po river, causes a state of eutrophication due to nutrient discharge [63]. Thus, it appears to be the key factor explaining the occurrence of high concentration of recruits of coastal species, such as red mullet, common Pandora and common sole in the central Northern Adriatic Sea.

Another large recruitment area occurs on the outer shelf of the central Adriatic Sea (GSA 17), southwards of the Pomo Pit, parallel to the Italian coast and in the South Adriatic Sea (GSA 18) on the northern side of the South Adriatic Pit. Finally, high density patches of recruits of several species were found in North Aegean Sea and Eastern Ionian Sea.

The co-occurrence of nurseries of different species along with the temporal persistence of these areas support the “ocean triad” hypothesis of Bakun [64] that links favourable spawning habitats to the combined effect of three main oceanographic mechanisms (1) enrichment (e.g. upwelling, water mixing), (2) concentration (convergence, frontal formation, water column stability) and (3) retention within (or drift toward) appropriate habitats. In the Tyrrhenian and Ligurian Seas, both eddies and frontal systems retain hake larvae and juveniles in areas of relatively high production [54], characterised by the occurrence of high biomass of macro-epibenthic suspension feeders, such as crinoids [11].

A more pertinent example is that of the Strait of Sicily where most of the identified nurseries are located along the eastern edge of the Adventure and Malta banks [27], [65]. Here, the Atlantic Ionian Stream, a surface current of Atlantic origin entering the Strait of Sicily west of Adventure Bank and flowing eastward, generates a number of semi-permanent features such as upwelling, eddies and fronts [66–67], which enhance and concentrate marine productivity [68]. In particular, the area east of Adventure Bank is characterized by the presence of a large cyclonic vortex, called the Adventure Bank Vortex which produces an upwelling at its centre that in turn increases primary and secondary production.

In GSA 18, the Southern Adriatic vortex produces an upwelling system, which enhances the productivity of the basin, while two main currents, the south-western, formed by colder and low salinity waters from the Po river and the south-eastern, carrying warmer and saltier waters from the eastern basin [69] contribute to the generation of favourable conditions for the occurrence of nursery areas. Those of thermophile and halophile species, such as the deep-water rose shrimp, are mainly localised along the eastern side, where nurseries of other coastal and deep-water species also occur, possibly as an effect of a lower fishing pressure.

In the Northern Adriatic Sea the nursery areas observed in the western coastal area are linked with the same north-west—south-east currents [70], while the middle Adriatic pit is an area where regional hydrography, low benthic biomass, and sedimentological factors combine, resulting in a nursery ground for hake and horned octopus.

Bakun’s ocean triad hypothesis cannot, however, be applied to elasmobranchs, such as the blackmouth catshark and thornback ray, producing benthic eggs. Nursery grounds of these species correspond to the areas where gravid females concentrate for hatching. From a conservation perspective the observed temporal stability of the identified nurseries of these two species implies a certain level of philopatry for their natal nursery grounds. This behaviour, furthermore, makes them even more susceptible to regional overfishing and localised stock depletion and extinction [71].

Our analyses of recruit distribution have demonstrated that the only nurseries consistently protected in European Mediterranean waters are those of coastal species, such as red mullet, common Pandora and common sole with 66.8%, 54.1% and 46.1% respectively of persistent nursery areas under protection. This is mostly due to the trawling ban within 3 nautical miles of the shoreline or 50 m depth, applied through current management measures as defined by Article 13 of EU Council Regulation 1967/2006. The proportion of nursery areas protected by ongoing conservation measures is much less consistent for species with offshore recruitment areas such as European hake and the deep-water rose shrimp, whose recruitment occurs on the shelf-break and upper slope below 150 m depth. Finally, there was minimal overlap between TFRA and recruitment areas for offshore species distributed over the continental slope deeper than 200 m (i.e. thornback ray, blackmouth catshark, giant red shrimp and Norway lobster). It is also worth noting that the overlaps seen here between spatial measures and persistent nurseries could represent an underestimate as the temporal extent of fishing closure can vary considerably in the Mediterranean. As a consequence the duration of closures might not always be sufficient, or at the appropriate time to coincide with recruitment periods.

Traditionally, Mediterranean fisheries have been oriented towards a multispecies complex, and the application of a mix of management tools based only on technical measures and fishing capacity control have mostly failed to ensure the long-term sustainability of fisheries, or the conservation of important habitats [3], [72]. We believe that the implementation of spatial management measures to protect areas where juveniles congregate during their first year of life has the potential to substantially improve current fisheries exploitation patterns. As observed by previous studies, the spatial closure of Mediterranean nurseries can yield important benefits to fisheries in terms of increases in resilience to fishing and yields [73–75]. In addition, it would allow for better compliance with the landing obligation implemented by the new Common Fisheries Policy, and a reduction in the high fishing mortality on juveniles that is currently undermining the productivity of Mediterranean demersal stocks [3], [6].

A combination of poorly selective gears (i.e. fine mesh sizes) and lack of spatial conservation measures currently leads to fisheries with high catches and discards of specimens below the MCRS, established for Mediterranean commercial stocks by the EC reg. 1967/2006 [4].

The new knowledge provided in this study on the distribution and persistence of demersal nurseries in EU Mediterranean waters can support the designation of no-take MPAs and their inclusion in a conservation network [27], [76–77]. Despite the fact that several spatial planning measures have been developed in the Mediterranean in the last few years to target different conservation objectives (see [78]), they have been only recently considered as management tools for fisheries [74], [77], [79]. In this context, our findings about the year-to-year stability of annual nursery areas in combination with the classification of each portion of the GSAs for their importance as recruitment areas, offer new opportunities to prioritize the management of marine space and, at the same time, facilitate provision of a greater-than-usual degree of risk aversion in management of activities in GSAs [80]. Whilst any future spatial conservation effort also needs to be evaluated for its socio-economic implications to minimize the conflict with resource users [77], [81], [82] it is important to point out that the establishment of a network of no-take areas in the Mediterranean at the moment appears to be one of the most straightforward ways to incorporate ecosystem conservation objectives into the management of demersal fisheries.

## Supporting Information

S1 Table Data source, methods applied and threshold lengths used for recruits identification of 11 commercial species of demersal fish and shellfish in Mediterranean geographical sub-areas (GSA). (DOCX)

S2 Table Summary of the models applied to map the annual distribution of recruits of demersal commercial species in Mediterranean FAO-GFCM Geographical Sub-Areas (GSAs). (DOCX)

S1 Text Cross validation indices used for model selection. (DOCX)
