# Supplementary material for: The Seascape of Demersal Fish Nursery Areas in the North Mediterranean Sea, a First Step Towards the Implementation of Spatial Planning for Trawl Fisheries
Source: PLoS One. 2015 Mar 18;10(3):e0119590. doi: 10.1371/journal.pone.0119590 (PMC4364973; doi:10.1371/journal.pone.0119590)
Supplement: S1 Table — (DOCX) [file pone.0119590.s001.docx]

| **S1 Table. Data source, methods applied and threshold lengths used for recruits identification of 11 commercial species of demersal fish and shellfish in Mediterranean geographical sub-areas (GSA).** | | | | |
| --- | --- | --- | --- | --- |
|  |  |  |  |  |
| **Species** | **GSAs** | **Data source** | **Method used** | **Mean threshold length (TL mm)** |
| ***Merluccius merluccius*** | 1 | MEDITS | Bhattacharya | 97 |
|  | 5 | MEDITS | Bhattacharya | 87 |
|  | 6 | MEDITS | Bhattacharya | 90 |
|  | 7 | DCF/MEDITS | Bhattacharya | 100 |
|  | 9 | Bartolino et al., 2008^[[1]](#footnote-1)^ | Fixed threshold length | 140 |
|  | 10 | MEDITS | Bhattacharya | 129 |
|  | 11 | MEDITS | Bhattacharya | 130 |
|  | 15, 16 | MEDITS | Bhattacharya | 145 |
|  | 17 | MEDITS | Bhattacharya | 132 |
|  | 18 | MEDITS | Bhattacharya | 127 |
|  | 19 | MEDITS | Bhattacharya | 125 |
|  | 20 | MEDITS | Bhattacharya | 100 |
|  | 22, 23 | MEDITS | Bhattacharya | 100 |
| ***Mullus barbatus*** | 17 | MEDITS | Bhattacharya | 71 |
|  | 18 | MEDITS | Bhattacharya | 86 |
| ***Pagellus erythrinus*** | 10 | MEDITS | Cut-off size calculated as mean length of individuals at maturity stage 1 in all the years. | 146 |
|  | 11 | MEDITS | Bhattacharya | 122.7 |
|  | 17 | MEDITS | Bhattacharya | 77 |
|  | 18 | MEDITS | Cut-off size calculated as mean length of individuals at maturity stage 1, years: 1995-2010 | 140 |
| ***Raja clavata*** | 11 | MEDITS | Fixed threshold length | 354 |
|  | 15, 16 | MEDITS | Fixed threshold length | 410 |
| ***Galeus melastomus*** | 1 | MEDITS | Bhattacharya | 186 |
|  | 5 | MEDITS | Bhattacharya | 192 |
|  | 6 | MEDITS | Bhattacharya | 201 |
|  | 7 | DCF/ MEDITS | Fixed threshold | 180 |
|  | 9 | DCF/ MEDITS | Bhattacharya | 180 |
|  | 10 | MEDITS | Cut-off size calculated as mean length of individuals at maturity stage 1 in all the years | 266 |
|  | 11 | MEDITS | Fixed threshold length | 250 |
|  | 15, 16 | MEDITS | Fixed threshold length | 285 |
|  | 17 | Relini et al., 1999^2^^[[2]](#footnote-2)^ | Fixed threshold length | 280 |
|  | 18 | MEDITS | Cut-off size calculated as mean length of individuals at maturity stage 1 in all the years | 271 |
|  | 19 | MEDITS | Bhattacharya | 175 |
| ***Solea solea*** | 17 | SoleMon survey | Bhattacharya | 190 |
| ***Aristaeomorpha foliacea*** | 1,5,6 | MEDITS | Fixed threshold length | 20 |
|  | 10 | MEDITS | Bhattacharya | 30 |
|  | 11 | MEDITS | Fixed threshold length | 28 |
|  | 15, 16 | MEDITS | Bhattacharya | 30 |
|  | 17 | Relini et al., 1999^2^ | Fixed threshold length | 30 |
|  | 18 | MEDITS | Bhattacharya | 31 |
|  | 19 | MEDITS | Bhattacharya | 25 |
|  | 20 | MEDITS | Bhattacharya | 23 |
|  | 22, 23 | MEDITS | Bhattacharya | 20 |
| **Species** | **GSAs** | **Data source** | **Method used** | **Mean threshold length (TL mm)** |
| ***Parapenaeus longirostris*** | 1 | MEDITS | Bhattacharya | 14.1 |
|  | 5 | MEDITS | Bhattacharya | 11.4 |
|  | 6 | MEDITS | Bhattacharya | 13.5 |
|  | 9 | DCF/MEDITS | Bhattacharya | 14.5 |
|  | 10 | MEDITS | Cut-off size calculated as average of Bhattacharya mean length from the years 1994, 1997, 1999, 2000, 2003, 2004 | 14.5 |
|  | 11 | MEDITS | Fixed threshold length | 16.2 |
|  | 15, 16 |  |  | 19.5 |
|  | 17 | MEDITS | Bhattacharya | 16 |
|  | 18 | MEDITS | Cut-off size calculated as average of Bhattacharya mean length from the years 1994, 1997, 1999, 2000, 2003, 2004 from GSA 10 | 14.5 |
|  | 19 | MEDITS | Bhattacharya | 20 |
|  | 20 | MEDITS | Bhattacharya | 10 |
|  | 22, 23 | MEDITS | Bhattacharya | 10 |
| ***Nephrops norvegicus*** | 1, 5, 6 | Orsi-Relini et al., 1998^[[3]](#footnote-3)^ |  | 13 |
|  | 7 | DCF/MEDITS | Maturity ogives | 27 |
|  | 8 | DCF/MEDITS | Maturity ogives | 30 |
|  | 9 | DCF/MEDITS | Fixed threshold length | 20 |
|  | 10 | MEDITS | L_25_ of the maturity ogive | 27 |
|  | 11 | MEDITS | Fixed threshold length | 25 |
|  | 15, 16 | DCF | Fixed threshold length | 26 |
|  | 17 | Piccinetti et al., 2012^[[4]](#footnote-4)^ | Fixed threshold length | 20 |
|  | 18 | MEDITS | L_25_ of the maturity ogive | 23 |
|  | 19 | MEDITS | Fixed threshold length | 20 |
|  | 20 | MEDITS | Bhattacharya | 25 |
|  | 22, 23 | MEDITS | Bhattacharya | 20 |
| ***Eledone cirrhosa*** | 1, 5, 6 | MEDITS | Fixed threshold length | 30 |
|  | 7 | DCF/MEDITS | Bhattacharya | 50 |
|  | 9 | DCF/MEDITS | Bhattacharya | 35 |
|  | 10 | MEDITS | Bhattacharya | 45 |
|  | 11 | MEDITS | Fixed threshold length | 37.6 |
|  | 18 | MEDITS | Bhattacharya | 53 |
|  | 19 | MEDITS | Fixed threshold length | 50 |
|  | 15, 16 | MEDITS | Bhattacharya | 48 |
|  | 17 | MEDITS | Bhattacharya | 43 |
|  | 20 | MEDITS | Bhattacharya | 40 |
|  | 22, 23 | MEDITS | Bhattacharya | 40 |
| ***Illex coindetii*** | 1 | MEDITS | Bhattacharya | 72 |
|  | 5 | MEDITS | Fixed threshold length | 62 |
|  | 6 | MEDITS | Bhattacharya | 69 |
|  | 9 | DCF/MEDITS | Bhattacharya | 60 |
|  | 10 | MEDITS | Bhattacharya | 90 |
|  | 11 | MEDITS | Fixed threshold length | 115 |
|  | 18 | MEDITS | Bhattacharya | 95 |
|  | 19 | MEDITS | Fixed threshold length | 90 |
|  | 15, 16 | MEDITS | Fixed threshold | 90 |
|  | 17 | Piccinetti et al., 2012^4^ | Fixed threshold length | 60 |
|  | 20 | MEDITS | Bhattacharya | 100 |
|  | 22, 23 | MEDITS | Bhattacharya | 100 |

1. Bartolino V., Ottavi A., Colloca F., Ardizzone G.D., Stefansson G. 2008. Bathymetric preferences in hake juveniles (Merluccius merluccius). ICES J. Mar. Sci. 65: 963-969. 228. [↑](#footnote-ref-1)
2. Relini G., Bertrand J., Zamboni A. (eds.). Syndem 1999. Synthesis of the knowledge on bottom fishery resources in Central Mediterranean Italy and Corsica. Biol Mar Medit., 6 (suppl. 1): 868 pp [↑](#footnote-ref-2)
3. Orsi Relini, L., Zamboni, A., Fiorentino, F. & Massi, D., 1998. Reproductive patterns in Norway lobster Nephrops norvegicus (L.), (Crustacea Decapoda: Nephropidae) of different Mediterranean areas. Sci. Mar. 62, 25–41 [↑](#footnote-ref-3)
4. Piccinetti C., Vrgoč N., Marčeta B., Manfredi C. 2012. Recent state of demersal resource in the Adriatic Sea. Acta Adriatica. Acta adriatica monograph series, 5. [↑](#footnote-ref-4)
