# Supplementary material for: The Seascape of Demersal Fish Nursery Areas in the North Mediterranean Sea, a First Step Towards the Implementation of Spatial Planning for Trawl Fisheries
Source: PLoS One. 2015 Mar 18;10(3):e0119590. doi: 10.1371/journal.pone.0119590 (PMC4364973; doi:10.1371/journal.pone.0119590)
Supplement: S2 Table — (DOCX) [file pone.0119590.s002.docx]

**S2 Table. Summary of the models applied to map the annual distribution of recruits of demersal commercial species in Mediterranean FAO-GFCM Geographical Sub-Areas (GSAs).**

**European hake: final models**

|  |  |  |  |  | Goodness of fit |  |  |  |
| --- | --- | --- | --- | --- | --- | --- | --- | --- |
| GSA | Period | Model Type |  | Covariates | Explained Deviance (%) | R^2^adj | R^2^ | CVI |
| 5 | 2007-2010 | ZIGAM: Presence-absence data | | year, depth, lat, lon | 59.2 | 0.68 |  |  |
|  |  | Non-zero data | | year, depth, lat, lon | 41.8 |  |  |  |
| 1, 6 | 1994-2010 | ZIGAM: Presence-absence data | | depth, lat, lon | 46.8 | 0.62 |  |  |
|  |  | Non-zero data | | year, depth, lat, lon | 36.2 |  |  |  |
| 7 | 1994-2010 | GAMM | | year, depth, distance from the coast + spatial component |  |  | 0.53 | 1.03 |
| 9 | 1994-2010 | GAMM | | year, depth, distance from the coast, bottom steepness + spatial component |  |  | 0.52 | 0.98 |
| 10 | 1994-2010 | ZIGAM: Presence-absence data | | depth + spatial component | 23.2 | 0.2 |  |  |
|  |  | Non-zero data | |  | 51.7 | 0.56 |  |  |
| 11 | 1994-2010 | ZIGAM: Presence-absence data | | depth + spatial component | 23.4 | 0.2 |  |  |
|  |  | Non-zero data | |  | 34.1 | 0.38 |  |  |
| 18 | 1996-2010 | ZIGAM: Presence-absence data | | depth + spatial component | 20.2 | 0.18 |  |  |
|  |  | Non-zero data | |  | 47.3 | 0.53 |  |  |
| 19 | 1994-2010 | ZIGAM: Presence-absence data | | depth + spatial component | 27 | 0.2 |  |  |
|  |  | Non-zero data | |  | 40.9 | 0.38 |  |  |
| 15, 16 | 2003-2010 | GAMM |  | year, distance from the coast, + spatial component |  |  | 0.58 | 0.99 |
| 17 | 2002-2010 | ZIGAM: Presence-absence data | | year, depth, lat, lon | 54.9 | 0.6 |  |  |
|  |  | Non-zero data | |  | 61.4 | 0.6 |  |  |
| 20 | 1999-2008 | ZIGAM: Presence-absence data | | year, depth, lat, lon | 50.7 | 0.51 |  |  |
|  |  | Non-zero data | |  | 47.7 | 0.36 |  |  |
| 22-23 | 1996-2008 | ZIGAM: Presence-absence data | | year, depth, lat, lon | 3.5 | 0.36 |  |  |
|  |  | Non-zero data | |  | 38.1 | 0.33 |  |  |

**European hake in GSAs 10, 11, 18, 19**. Parameters of ordinary kriging applied on the residuals of ZIGAM models.

A pooled empirical variogram was computed by taking the mean of all annual semi-variances within each lag class (Morfin et al., 2012)

| GSA | Covariance function | Range (km) | Sill | Nugget | CVI |
| --- | --- | --- | --- | --- | --- |
| 10 | Gaussian | 7.04 | 8.49E+06 | 2.81E+06 | 0.31 |
| 11 | Exponential | 19.50 | 1.70E+07 | 2.74E+06 | 1.42 |
| 18 | Gaussian | 12.25 | 1.29E+06 | 2.75E+05 | 0.69 |
| 19 | Exponential | 24.23 | 9.29E+04 | 3.32E+04 | 0.67 |

**European hake in GSA 7***.* Parameters of ordinary kriging applied on GAM residuals

| GSA | Year | Model Type | Covariance function | Nugget | Sill | Range (km) |
| --- | --- | --- | --- | --- | --- | --- |
| 7 | 1994 | Ordinary Kriging | Spherical | 0.06 | 4.60 | 6.75 |
|  | 1995 |  | Spherical | 0 | 7.03 | 8.89 |
|  | 1997 |  | Exponential | 0 | 3.35 | 12.22 |
|  | 1998 |  | Exponential | 0 | 3.15 | 1.03 |
|  | 1999 |  | Exponential | 0.12 | 3.38 | 0.33 |
|  | 2000 |  | Spherical | 1 | 1.80 | 0.65 |
|  | 2001 |  | Exponential | 0.0 | 2.38 | 9.37 |
|  | 2002 |  | Spherical | 0 | 3.01 | 16.70 |
|  | 2003 |  | Spherical | 1.7 | 3.07 | 19.88 |
|  | 2004 |  | Exponential | 0.5 | 1.57 | 1.81 |
|  | 2005 |  | Spherical | 0 | 3.53 | 6.03 |
|  | 2006 |  | Spherical | 0.63 | 1.45 | 38.45 |
|  | 2007 |  | Exponential | 1.2 | 2.66 | 11.90 |
|  | 2008 |  | Spherical | 1.455 | 1.164 | 2 |
|  | 2009 |  | Spherical | 0.343 | 2.991 | 4.775 |
|  | 2010 |  | Spherical | 0.897 | 4.483 | 5 |

**European hake in GSA 9***.* Parameters of ordinary kriging applied on GAM residuals

| GSA | Year | Model Type | Covariance function | Nugget | Sill | Range (km) |
| --- | --- | --- | --- | --- | --- | --- |
| 9 | 1994 | Ordinary Kriging | Spherical | 4.24 | 3.66 | 30.97 |
|  | 1995 |  | Spherical | 2.43 | 6.26 | 29.44 |
|  | 1996 |  | Spherical | 0.65 | 8.35 | 19.32 |
|  | 1997 |  | Spherical | 2.97 | 7.37 | 31.61 |
|  | 1998 |  | Spherical | 1.16 | 10.15 | 16.33 |
|  | 1999 |  | Spherical | 1.03 | 9.48 | 18.43 |
|  | 2000 |  | Spherical | 2.50 | 6.16 | 25.56 |
|  | 2001 |  | Spherical | 0.00 | 8.71 | 12.05 |
|  | 2002 |  | Spherical | 0.00 | 10.62 | 18.04 |
|  | 2003 |  | Spherical | 0.00 | 8.76 | 17.09 |
|  | 2004 |  | Spherical | 1.99 | 7.98 | 18.71 |
|  | 2005 |  | Spherical | 0.00 | 9.13 | 8.48 |
|  | 2006 |  | Spherical | 5.32 | 3.73 | 32.70 |
|  | 2007 |  | Spherical | 1.15 | 7.91 | 8.13 |
|  | 2008 |  | Spherical | 2.06 | 7.93 | 24.95 |
|  | 2009 |  | Spherical | 0.00 | 11.17 | 17.10 |
|  | 2010 |  | Spherical | 0.897 | 4.483 | 5 |

**Red mullet: final models**

|  |  |  |  |  | Goodness of fit |  | |  |
| --- | --- | --- | --- | --- | --- | --- | --- | --- |
| GSA | Period | Model Type |  | Covariates | Explained Deviance (%) | R^2^adj | |  |
| 17 | 2002-2008 | ZIGAM: Presence-absence data | | depth, year, lon, lat | 41.8 | 0.31 | |  |
|  |  | Non-zero data | |  | 54.8 | 0.42 | |  |
|  |  |  |  |  |  |  |  |  |
| GSA | Period | Model Type |  | *Covariance function | Range (km) | Sill | Nugget | CVI |
| 18 | 1999,2005, 2007 | Ordinary kriging: |  | Gaussian | 15.46 | 1.23E+07 | 4.08E+06 | 0.01 |
|  |  |  | |  |  |  |  |  |

* pooled empirical variogram was computed by taking the mean of all annual semi-variances within each lag class (Morfin et al., 2012)

**Common pandora: final models**

|  |  |  |  |  | Goodness of fit |  | |  |
| --- | --- | --- | --- | --- | --- | --- | --- | --- |
| GSA | Period | Model Type |  | Covariates | Explained Deviance (%) | R^2^adj | |  |
| 17 | 2002-2008 | ZIGAM: Presence-absence data | | depth, year, lon, lat | 31.3 | 0.18 | |  |
|  |  | Non-zero data | |  | 33.1 | 0.23 | |  |
|  |  |  |  |  |  |  |  |  |
| GSA | Period | Model Type | | *Covariance function | Range (km) | Sill | Nugget | CVI |
| 10 | 2002-2008 | Ordinary kriging | | Gaussian | 20.98 | 3.61E+05 | 1.00E-02 | 0.01 |
| 11 | 1994-2010 | Ordinary kriging | | Spherical | 20.98 | 3.61E+05 | 1.00E-02 | 3.18 |
| 18 | 1996-2010 | Ordinary kriging | | Gaussian | 11.58 | 1.42E+04 | 4.85E+02 | 0.47 |

* pooled empirical variogram was computed by taking the mean of all annual semi-variances within each lag class (Morfin et al., 2012)

**Thornback ray**: **final models**

|  |  |  |  |  | Goodness of fit |  | |  |  |
| --- | --- | --- | --- | --- | --- | --- | --- | --- | --- |
| GSA | Period | Model Type |  | Covariates | Explained Deviance (%) | R^2^adj | | R^2^ | CVI |
| 11 | 1994-2010 | ZIGAM: Presence-absence data | | + spatial component | 9.48 | 0.05 | |  |  |
|  |  | Non-zero data | |  | 22.24 | 0.19 | |  |  |
| 15, 16 | 2003-2010 | GLMM |  | - |  |  |  | 0.39 | 1.56 |

**Thornback ray** **in GSAs 15-16**. Parameters of ordinary kriging applied on the residuals of ZIGAMs.

| GSA | Year | Model Type | Covariance function | Nugget | Sill | Range (km) |
| --- | --- | --- | --- | --- | --- | --- |
| 15, 16 | 2003 | Ordinary Kriging | Exponential | 0.56 | 1.45 | 16.83 |
|  | 2004 |  | Exponential | 1.25 | 0.41 | 45.36 |
|  | 2005 |  | Exponential | 0.85 | 0.97 | 30.22 |
|  | 2006 |  | Exponential | 1.48 | 0.99 | 84.82 |
|  | 2007 |  | Exponential | 0.00 | 1.23 | 18.20 |
|  | 2008 |  | Exponential | 0.00 | 2.30 | 14.11 |
|  | 2009 |  | Exponential | 0.00 | 1.25 | 9.73 |
|  | 2010 |  | Exponential | 0.00 | 1.42 | 23.61 |

**Blackmouth catshark: final models**

|  |  |  |  |  | Goodness of fit |  |  |  |
| --- | --- | --- | --- | --- | --- | --- | --- | --- |
| GSA | Period | Model Type |  | Covariates | Explained Deviance (%) | R^2^adj | R^2^ | CVI |
| 5 | 2007-2010 | ZIGAM: Presence-absence data | | year, depth | 45.4 | 0.63 |  |  |
|  |  | Non-zero data | |  | 39 |  |  |  |
| 1, 6 | 2000-2010 | ZIGAM: Presence-absence data | | year, depth, lon, lat | 26.2 | 0.28 |  |  |
|  |  | Non-zero data | |  | 35.5 |  |  |  |
| 7 | 2003-2009 | GAM | | year, lat |  |  | 0.42 | 1.05 |
| 9 | 1999-2010 | Bayesian GLMM |  |  |  |  | 0.84 | 0.5 |
| 10 | 1999-2010 | ZIGAM: Presence-absence data | | Depth + spatial component | 43.33 | 0.41 |  |  |
|  |  | Non-zero data | |  | 19.31 | 0.16 |  |  |
| 11 | 1999-2010 | ZIGAM: Presence-absence data | | depth+ spatial component | 60.18 | 0.59 |  |  |
|  |  | Non-zero data | |  | 21.78 | 0.17 |  |  |
| 15, 16 | 2003-2010 | GLMM |  | - |  |  | 0.61 | 0.99 |
| 18 | 1999:2002, 2004:2010 | ZIGAM: Presence-absence data | | depth | 32.2 | 0.32 |  |  |
|  |  | Non-zero data | |  | 5.6 | 0.1 |  |  |
| 19 | 1995-2010 | ZIGAM: Presence-absence data | | depth | 18.5 | 0.2 |  |  |
|  |  | Non-zero data | |  | 29.2 | 0.1 |  |  |

**Blackmouth catshark in GSAs 10, 11, 18, 19**. Parameters of ordinary kriging applied on the residuals of ZIGAMs.

A pooled empirical variogram was computed by taking the mean of all annual semi-variances within each lag class (Morfin et al., 2012)

| GSA | Covariance function | Range (km) | Sill | Nugget | CVI |
| --- | --- | --- | --- | --- | --- |
| 10 | Gaussian | 15.84 | 4.37E+04 | 1.82E+04 | 4.47 |
| 11 | Exponential | 12.52 | 1.26E+06 | 6.01E+03 | 5.02 |
| 18 | Exponential | 11.01 | 1.00E+04 | 3.62E+03 | 3.10 |
| 19 | Exponential | 24.23 | 9.29E+04 | 3.32E+04 | 0.67 |

**Blackmouth catshark in GSA 9***.* Parameters of ordinary kriging applied on GLM residuals

| GSA | Year | Model Type | Covariance function | Nugget | Sill | Range (km) |
| --- | --- | --- | --- | --- | --- | --- |
| 9 | 1999 | Ordinary Kriging | Spherical | 0.415 | 2.04 | 32.788 |
|  | 2000 |  | Spherical | 0.676 | 2.201 | 35.528 |
|  | 2001 |  | Spherical | 0.625 | 2.168 | 36.049 |
|  | 2002 |  | Spherical | 0.874 | 1.707 | 41.997 |
|  | 2003 |  | Spherical | 0.547 | 1.698 | 91.262 |
|  | 2004 |  | Spherical | 0.696 | 1.539 | 48.51 |
|  | 2005 |  | Spherical | 0.727 | 1.14 | 19.714 |
|  | 2006 |  | Spherical | 0.603 | 2.176 | 54.905 |
|  | 2007 |  | Spherical | 0 | 2.631 | 7.985 |
|  | 2008 |  | Spherical | 0.964 | 2.062 | 27.416 |
|  | 2009 |  | Spherical | 1.119 | 1.371 | 63.757 |
|  | 2010 |  | Spherical | 1.096 | 1.494 | 60.181 |

**Blackmouth catshark in GSA 15-16***.* Parameters of ordinary kriging applied on GLM residuals

| GSAs | Year | Model Type | Covariance function | Nugget | Sill | Range (km) |
| --- | --- | --- | --- | --- | --- | --- |
| 15-16 | 2003 | Ordinary Kriging | Exponential | 1.03 | 2.46 | 38.43 |
|  | 2004 |  | Spherical | 1.57 | 2.33 | 40.40 |
|  | 2005 |  | Spherical | 0.94 | 3.36 | 43.33 |
|  | 2006 |  | Spherical | 2.18 | 2.13 | 107.37 |
|  | 2007 |  | Spherical | 1.67 | 3.49 | 86.42 |
|  | 2008 |  | Spherical | 0.82 | 5.26 | 65.12 |
|  | 2009 |  | Spherical | 1.40 | 5.24 | 127.64 |
|  | 2010 |  | Exponential | 1.79 | 3.32 | 72.15 |

**Common sole: final models**

| GSA | Year | Model Type | Covariance function | lag | Nugget | Sill | Range (km) | |
| --- | --- | --- | --- | --- | --- | --- | --- | --- |
| 17 | 2005 | Ordinary Kriging | Spherical | 3700 | 0.05 | 0.09 | 18.52 |  |
|  | 2006 |  | Spherical | 3700 | 0.05 | 0.04 | 18.52 |  |
|  | 2007 |  | Exponential | 3700 | 0.05 | 0.07 | 13.89 |  |
|  | 2008 |  | Spherical | 3700 | 0.05 | 0.05 | 14.816 |  |
|  | 2009 |  | Spherical | 3700 | 0.71 | 0.05 | 18.52 |  |
|  | 2010 |  | Spherical | 3700 | 0 | 0.13 | 16.668 |  |

**Deep-water rose shrimp: final models**

|  |  |  |  |  | Goodness of fit |  |  |  |
| --- | --- | --- | --- | --- | --- | --- | --- | --- |
| GSA | Period | Model Type |  | Covariates | Explained Deviance (%) | R^2^adj | R^2^ | CVI |
| 1, 6 | 1995-2004, 2006-2007, 2009-2010 | ZIGAM: Presence-absence data | | year, depth, lat, lon | 29 | 0.21 |  |  |
|  |  | Non-zero data | | year, depth, | 31.8 |  |  |  |
| 9 | 1994-2010 | Bayesian GLMM | | lat, bottom steepness + spatial component |  |  | 0.87 | 0.49 |
| 10 | 1994-2010 | ZIGAM: Presence-absence data | | depth + spatial component | 30.7 | 0.34 |  |  |
|  |  | Non-zero data | |  | 26.1 | 0.23 |  |  |
| 11 | 1995-2010 | ZIGAM: Presence-absence data | | depth + spatial component | 28.1 | 0.18 |  |  |
|  |  | Non-zero data | |  | 31.6 | 0.2 |  |  |
| 19 | 1996-2010 | ZIGAM: Presence-absence data | | depth + spatial component | 52.8 | 0.52 |  |  |
|  |  | Non-zero data | |  | 17.9 | 0.15 |  |  |
| 15, 16 | 2003-2010 | GLMM |  | year, distance from the coast, + spatial component |  |  | 0.65 | 0.99 |
| 17 | 2002-2010 | ZIGAM: Presence-absence data | | year, depth, lat, lon | 63.9 | 0.49 |  |  |
|  |  | Non-zero data | |  | 57.3 | 0.64 |  |  |
| 22-23 | 1996-2008 | ZIGAM: Presence-absence data | | year, depth, lat, lon | 63 | 0.58 |  |  |
|  |  | Non-zero data | |  |  |  |  |  |

| GSA | Period | Model Type | *Covariance function | Range (km) | Sill | Nugget | CVI |
| --- | --- | --- | --- | --- | --- | --- | --- |
| 18 | 1999-2002 | Ordinary kriging | Exponential | 17.85 | 5.60E+04 | 8.77E+02 | 1.81 |
|  | 2004-2010 |  |  |  |  |  |  |

*Residuals modelled using the Inverse Distance Weighting with 5 as power coefficient

**Deep-water rose shrimp in GSAs 10, 11, 18, 19**. Parameters of ordinary kriging applied on the residuals of ZIGAMs.

A pooled empirical variogram was computed by taking the mean of all annual semi-variances within each lag class (Morfin et al., 2012)

| GSA | Covariance function | Range (km) (km) | Sill | Nugget | CVI |
| --- | --- | --- | --- | --- | --- |
| 10 | Exponential | 14.46 | 1.73E+05 | 9.87E+04 | 0.57 |
| 11 | Exponential | 38.10 | 1.12E+04 | 9.59E+02 | 1.71 |
| 19 | Spherical | 54.48 | 2.11E+06 | 6.49E+05 | 1.83 |

**Deep-water rose shrimp in GSA 9***.* Parameters of ordinary kriging applied on residuals of Bayesian GLM

| GSA | Year | Model Type | Covariance function | Nugget | Sill | Range (km) |
| --- | --- | --- | --- | --- | --- | --- |
| 9 | 1996 | Ordinary kriging | Spherical | 1.23 | 0.88 | 12.65 |
|  | 1997 |  | Spherical | 0.45 | 1.67 | 15.25 |
|  | 1998 |  | Spherical | 0.36 | 1.62 | 15.38 |
|  | 1999 |  | Spherical | 1.03 | 1.61 | 15.19 |
|  | 2000 |  | Spherical | 0.91 | 1.58 | 14.94 |
|  | 2001 |  | Spherical | 0.34 | 1.46 | 14.16 |
|  | 2002 |  | Spherical | 2.45 | 1.49 | 14.30 |
|  | 2003 |  | Spherical | 2.60 | 1.50 | 14.35 |
|  | 2004 |  | Spherical | 0.41 | 1.47 | 14.13 |
|  | 2005 |  | Spherical | 2.84 | 1.46 | 14.09 |
|  | 2006 |  | Spherical | 0.22 | 1.28 | 12.90 |
|  | 2007 |  | Spherical | 0.82 | 1.32 | 13.20 |
|  | 2008 |  | Spherical | 0.92 | 1.29 | 13.02 |
|  | 2009 |  | Spherical | 3.49 | 1.32 | 13.20 |
|  | 2010 |  | Spherical | 4.42 | 1.34 | 13.37 |

**Deep-water rose shrimp in GSA 15-16***.* Parameters of ordinary kriging applied on GLM residuals

| GSA | Year | Model Type | Covariance function | Nugget | Sill | Range (km) |
| --- | --- | --- | --- | --- | --- | --- |
| 15-16 | 2003 | Ordinary kriging | Spherical | 3.21 | 10.9 | 54.90 |
|  | 2004 |  | Spherical | 0 | 13.5 | 16.05 |
|  | 2005 |  | Spherical | 1.24 | 8.21 | 20.88 |
|  | 2006 |  | Spherical | 1.95 | 8.96 | 18.00 |
|  | 2007 |  | Spherical | 0 | 7.44 | 20.95 |
|  | 2008 |  | Spherical | 0 | 12.26 | 23.86 |
|  | 2009 |  | Spherical | 0 | 11.55 | 22.71 |
|  | 2010 |  | Spherical | 0 | 10.45 | 23.07 |

**Norway lobster: final models**

|  |  |  |  |  | Goodness of fit |  |  |  |
| --- | --- | --- | --- | --- | --- | --- | --- | --- |
| GSA | Period | Model Type |  | Covariates | Explained Deviance (%) | R^2^adj | R^2^ | CVI |
|  |  | Non-zero data | |  | 40.9 | 0.38 |  |  |
| 15, 16 | 2003-2010 | GLMM |  | spatial component |  |  | 0.38 | 0.99 |
| 17 | 2002-2010 | ZIGAM: Presence-absence data | | year, depth, lat, lon | 53.9 | 0.43 |  |  |
|  |  | Non-zero data | |  | 67.3 | 0.54 |  |  |

| GSA | Period | Model Type | *Covariance function | Range (km) (km) | Sill | Nugget | CVI |
| --- | --- | --- | --- | --- | --- | --- | --- |
| 19 | 1994-2010 | Ordinary kriging | Spherical | 73.5 | 3.17E+02 | 1.79E+02 | 0.20 |
| 11 | 1999-2001  2003-2010 | Ordinary kriging | Spherical | 28.3 | 1.99E+03 | 4.44E+02 | 0.44 |
|  |  |  |  |  |  |  |  |

**Norway lobster in GSA 15-16***.* Parameters of ordinary kriging applied on GLM residuals

| GSA | Year | Model Type | Covariance function | Nugget | Sill | Range (km) |
| --- | --- | --- | --- | --- | --- | --- |
| 15-16 | 2003 | Ordinary kriging | Spherical | 0.68 | 0.27 | 27.04 |
|  | 2004 |  | Spherical | 1.00 | 1.46 | 78.16 |
|  | 2005 |  | Spherical | 0.00 | 2.23 | 12.64 |
|  | 2006 |  | Exponential | 1.81 | 1.04 | 15.66 |
|  | 2007 |  | Spherical | 1.86 | 1.65 | 14.66 |
|  | 2008 |  | Spherical | 0.54 | 3.91 | 27.07 |
|  | 2009 |  | Spherical | 0.50 | 3.98 | 33.74 |
|  | 2010 |  | Spherical | 0.99 | 2.87 | 20.62 |

**Giant red shrimp: final models**

|  |  |  |  |  | Goodness of fit |  |  |  |
| --- | --- | --- | --- | --- | --- | --- | --- | --- |
| GSA | Period | Model Type |  | Covariates | Explained Deviance (%) | R^2^adj | R^2^ | CVI |
| 15-16 | 2003-2010 | GAMM | | lat, distance from the coast + spatial component |  |  | 0.43 | 0.95 |
| 10 | 1994-2010 | ZIGAM: Presence-absence data | | depth + spatial component | 35.54 | 0.41 |  |  |
|  |  | Non-zero data | |  | 16.11 | 0.11 |  |  |
| 11 | 1994-2010 | ZIGAM: Presence-absence data | | depth + spatial component | 37.8 | 0.39 |  |  |
|  |  | Non-zero data | |  | 9.48 | 0.10 |  |  |
| 18 | 1996:2006  2008:2010 | ZIGAM: Presence-absence data | | depth + spatial component | 32.25 | 0.30 |  |  |
|  |  | Non-zero data | |  | 12.72 | 0.07 |  |  |
| 19 | 2002-2010 | ZIGAM: Presence-absence data | | year, depth, lat, lon | 25.0 | 0.20 |  |  |
|  |  | Non-zero data | |  | 41.6 | 0.40 |  |  |

**Giant red shrimp in GSAs 10, 11, 18, 19.** Parameters of ordinary kriging applied on the residuals of ZIGAM models.

A pooled empirical variogram was computed by taking the mean of all annual semi-variances within each lag class (Morfin et al., 2012)

| GSA | Covariance function | Range (km) | Sill | Nugget | CVI |
| --- | --- | --- | --- | --- | --- |
| 10 | Gaussian | 17.85 | 5.08E+06 | 1.52E+06 | 2.01 |
| 11 | Gaussian | 34.67 | 3.00E+06 | 1.30E+06 | 0.52 |
| 18 | Exponential | 6.56 | 4.67E+04 | 1.33E+03 | 0.03 |
| 19 | Gaussian | 68.29 | 9.84E+04 | 5.07E+04 | 0.05 |

**Giant red shrimp in GSA 15-16***.* Parameters of ordinary kriging applied on GLM residuals

| GSA | Year | Model Type | Covariance function | Nugget | Sill | Range (km) |
| --- | --- | --- | --- | --- | --- | --- |
| 15-16 | 2003 | Ordinary kriging | Spherical | 0 | 3.67 | 17.48 |
|  | 2004 |  | Spherical | 0.27 | 3.56 | 20.45 |
|  | 2005 |  | Spherical | 0 | 3.74 | 10.25 |
|  | 2006 |  | Spherical | 0 | 2.03 | 13.09 |
|  | 2007 |  | Exponential | 0.11 | 3.55 | 8.16 |
|  | 2008 |  | Spherical | 0 | 3.17 | 8.07 |
|  | 2009 |  | Spherical | 0 | 2.74 | 12.63 |
|  | 2010 |  | Spherical | 0 | 1.99 | 19.71 |

**Horned octopus: final models**

|  |  |  |  |  | Goodness of fit |  |  |  |
| --- | --- | --- | --- | --- | --- | --- | --- | --- |
| GSA | Period | Model Type |  | Covariates | Explained Deviance (%) | R^2^adj | R^2^ | CVI |
| 7 | 1994-2009 | GAMM | | year, distance from the coast , bottom steepness + spatial component |  |  | 0.51 | 1.03 |
| 9 | 1994-2010 | GAMM | | year, depth, distance from the coast, bottom steepness + spatial component |  |  | 0.38 | 1.01 |
| 10 | 1994-2010 | ZIGAM: Presence-absence data | | depth + spatial component | 27.88 | 0.23 |  |  |
|  |  | Non-zero data | |  | 35.87 | 0.3 |  |  |
| 11 | 1994-2010 | ZIGAM: Presence-absence data | | depth + spatial component | 23.41 | 0.28 |  |  |
|  |  | Non-zero data | |  | 15.11 | 0.12 |  |  |
| 18 | 1996-2010 | ZIGAM: Presence-absence data | | depth + spatial component | 19.55 | 0.14 |  |  |
|  |  | Non-zero data | |  | 24.25 | 0.18 |  |  |
| 19 | 1994-2010 | ZIGAM: Presence-absence data | | depth + spatial component* | 11.2 | 0.1 |  |  |
|  |  | Non-zero data | |  | 19.55 | 0.14 |  |  |
| 17 | 2002-2010 | ZIGAM: Presence-absence data | | year, depth, lat, lon | 40.8 | 0.38 |  |  |
|  |  | Non-zero data | |  | 52.7 | 0.47 |  |  |
|  |  | Non-zero data | |  | 47.7 | 0.36 |  |  |
| 22-23 | 1998-2008 | ZIGAM: Presence-absence data | | year, depth, lat, lon | 47.9 | 0.40 |  |  |
|  |  | Non-zero data | |  |  |  |  |  |

***Residuals modelled using the Inverse Distance Weighting with 5 as power coefficient

**Horned octopus in GSAs 10, 11, 18, 19.** Parameters of ordinary kriging applied on the residuals of ZIGAMs.

A pooled empirical variogram was computed by taking the mean of all annual semi-variances within each lag class (Morfin et al., 2012)

| GSA | Covariance function | Range (km) | Sill | Nugget | CVI |
| --- | --- | --- | --- | --- | --- |
| 10 | Spherical | 15.13 | 3.36E+03 | 1.27E+03 | 0.14 |
| 11 | Exponential | 17.78 | 1.74E+04 | 6.89E+03 | 0.43 |
| 18 | Exponential | 9.34 | 8.19E+03 | 4.58E+02 | 0.10 |
| 19 | Gaussian | 68.29 | 9.84E+04 | 5.07E+04 | 0.05 |

**Horned octopus in GSA 9***.* Parameters of ordinary kriging applied on the GAM residuals

| GSA | Year | Model Type | Covariance function | Nugget | Sill | Range (km) |
| --- | --- | --- | --- | --- | --- | --- |
| 9 | 1994 | Ordinary kriging | Spherical | 1.07 | 2.08 | 25.17 |
|  | 1995 |  | Spherical | 1.59 | 1.57 | 29.80 |
|  | 1996 |  | Spherical | 1.98 | 1.02 | 30.98 |
|  | 1997 |  | Spherical | 2.17 | 1.06 | 35.30 |
|  | 1998 |  | Spherical | 0.00 | 3.07 | 8.11 |
|  | 1999 |  | Spherical | 0.68 | 1.69 | 26.60 |
|  | 2000 |  | Spherical | 0.00 | 2.64 | 16.49 |
|  | 2001 |  | Spherical | 1.95 | 1.75 | 30.09 |
|  | 2002 |  | Spherical | 0.00 | 3.11 | 14.84 |
|  | 2003 |  | Spherical | 0.00 | 3.11 | 10.61 |
|  | 2004 |  | Spherical | 0.50 | 2.33 | 18.89 |
|  | 2005 |  | Spherical | 2.05 | 0.99 | 74.66 |
|  | 2006 |  | Spherical | 1.27 | 1.24 | 25.73 |
|  | 2007 |  | Spherical | 0.00 | 2.58 | 13.41 |
|  | 2008 |  | Spherical | 0.26 | 1.99 | 10.00 |
|  | 2009 |  | Spherical | 1.52 | 0.98 | 26.40 |
|  | 2010 |  | Spherical | 1.07 | 2.08 | 25.17 |

**Horned octopus in GSA 7***.* Parameters of ordinary kriging applied on GAM residuals

| GSA | Year | Model Type | Covariance function | Nugget | Sill | Range (km) |
| --- | --- | --- | --- | --- | --- | --- |
| 7 | 1994 | Ordinary kriging | Spherical | 0.00 | 3.66 | 19.03 |
|  | 1997 |  | Spherical | 0.17 | 2.01 | 24.41 |
|  | 1998 |  | Spherical | 0.30 | 2.31 | 8.03 |
|  | 1999 |  | Spherical | 0.00 | 2.06 | 12.57 |
|  | 2000 |  | Spherical | 0.12 | 3.52 | 19.05 |
|  | 2001 |  | Spherical | 0.00 | 4.45 | 17.05 |
|  | 2003 |  | Spherical | 1.15 | 1.36 | 34.21 |
|  | 2004 |  | Spherical | 1.60 | 1.66 | 49.46 |
|  | 2005 |  | Spherical | 0.00 | 2.58 | 21.74 |
|  | 2007 |  | Spherical | 2.13 | 3.37 | 31.40 |
|  | 2008 |  | Spherical | 0.00 | 3.07 | 9.68 |
|  | 2009 |  | Spherical | 0.00 | 4.32 | 6.30 |

**Broadtail shortfin squid: final models**

|  |  |  |  |  | Goodness of fit |  |  |  |
| --- | --- | --- | --- | --- | --- | --- | --- | --- |
| GSA | Period | Model Type |  | Covariates | Explained Deviance (%) | R^2^adj | R^2^ | CVI |
| 9 | 1994-2010 | GAMM | | distance from the coast + spatial component |  |  | 0.39 | 1.01 |
| 15-16 | 2003-2010 | GAMM | | lat, lon+ spatial component |  |  | 0.46 | 0.96 |
|  | 1995-1997, 1999-2006, 2008-2010 | ZIGAM: Presence-absence data  Non-zero data | | year, lat, lon, depth | 26.8 | 0.31 |  |  |
| 1-6 |  |  |  |  | 24.5 |  |  |  |
| 10 | 1994-2010 | ZIGAM: Presence-absence data | | depth + spatial component | 31.73 | 0.33 |  |  |
|  |  | Non-zero data | |  | 23.55 | 0.18 |  |  |
| 11 | 1994-2010 | ZIGAM: Presence-absence data | | depth + spatial component | 30.72 | 0.3 |  |  |
|  |  | Non-zero data | |  | 20.67 | 0.17 |  |  |
| 18 | 1996-2010 | ZIGAM: Presence-absence data | | depth + spatial component | 28.12 | 0.33 |  |  |
|  |  | Non-zero data | |  | 17.07 | 0.14 |  |  |
| 19 | 1994-2010 | ZIGAM: Presence-absence data | | depth + spatial component* | 33.0 | 0.3 |  |  |
|  |  | Non-zero data | |  | 20.9 | 0.2 |  |  |
| 20 | 1998-2008 | ZIGAM: Presence-absence data | | year, depth, lat, lon | 52.3 | 0.45 |  |  |
|  |  | Non-zero data | |  |  |  |  |  |

| GSA | Year | Model Type | Model | lag | Nugget | Sill | Range (km) | |
| --- | --- | --- | --- | --- | --- | --- | --- | --- |
| 17 | 2002 | Ordinary Kriging | Exponential | 18520 | 0 | 1174755 | 101415 |  |
|  | 2003 |  | Spherical | 18520 | 4444 | 16835 | 35478 |  |
|  | 2004 |  | Exponential | 18520 | 68579 | 145327 | 253044 |  |
|  | 2005 |  | Exponential | 18520 | 3396 | 36080 | 32057 |  |
|  | 2006 |  | Spherical | 3000 | 15344 | 40264 | 4244 |  |
|  | 2007 |  | Spherical | 9000 | 0 | 278989 | 18245 |  |
|  | 2008 |  | Exponential | 9000 | 13831 | 18934 | 54346 |  |
|  | 2009 |  | Exponential | 18520 | 0 | 14696 | 116820 |  |
|  | 2010 |  | Spherical | 18520 | 8593 | 4525 | 37899 |  |

**Broadtail shortfin squid in GSAs 10, 11, 18, 19.** Parameters of ordinary kriging applied on the residuals of ZIGAMs.

A pooled empirical variogram was computed by taking the mean of all annual semi-variances within each lag class (Morfin et al., 2012)

| GSA | Covariance function | Range (km) | Sill | Nugget | CVI |
| --- | --- | --- | --- | --- | --- |
| 10 | Gaussian | 8.46 | 1.09E+06 | 1.99E+04 | 0.01 |
| 11 | Exponential | 18.59 | 2.25E+05 | 1.40E+04 | 0.02 |
| 18 | Spherical | 39.52 | 1.08E+06 | 3.67E+05 | 0.26 |
| 19 | Spherical | 15.77 | 8.13E+04 | 4.12E+03 | 1.28 |

**Broadtail shortfin squid in GSA 9***.* Parameters of ordinary kriging applied on GAM residuals

| GSA | Year | Model Type | Covariance function | Nugget | Sill | Range (km) |
| --- | --- | --- | --- | --- | --- | --- |
| 9 | 1994 | Ordinary kriging | Spherical | 1.53 | 0.20 | 31.73 |
|  | 1995 |  | Spherical | 1.96 | 1.06 | 28.63 |
|  | 1996 |  | Spherical | 0.00 | 3.93 | 13.82 |
|  | 1997 |  | Exponential | 0.11 | 4.76 | 17.30 |
|  | 1998 |  | Spherical | 2.29 | 1.62 | 25.43 |
|  | 1999 |  | Spherical | 1.16 | 1.05 | 24.54 |
|  | 2000 |  | Spherical | 0.55 | 2.71 | 8.29 |
|  | 2001 |  | Spherical | 1.30 | 0.95 | 22.62 |
|  | 2002 |  | Spherical | 3.60 | 2.54 | 36.75 |
|  | 2003 |  | Spherical | 0.52 | 2.01 | 18.11 |
|  | 2004 |  | Spherical | 0.50 | 0.63 | 18.07 |
|  | 2005 |  | Spherical | 0.00 | 6.09 | 7.95 |
|  | 2006 |  | Spherical | 0.00 | 2.79 | 22.62 |
|  | 2007 |  | Spherical | 0.00 | 5.08 | 7.14 |
|  | 2008 |  | Spherical | 0.00 | 2.33 | 14.15 |
|  | 2009 |  | Spherical | 1.53 | 0.20 | 31.73 |
|  | 2010 |  | Spherical | 1.96 | 1.06 | 28.63 |

**Broadtail shortfin squid in GSA 15-16***.* Parameters of ordinary kriging applied on the residuals of GAM

| GSA | Year | Model Type | Covariance function | Nugget | Sill | Range (km) |
| --- | --- | --- | --- | --- | --- | --- |
| 15-16 | 2003 | Ordinary kriging | Spherical | 0.00 | 6.68 | 20.49 |
|  | 2004 |  | Spherical | 0.00 | 4.23 | 14.55 |
|  | 2005 |  | Spherical | 1.12 | 1.16 | 15.61 |
|  | 2006 |  | Spherical | 1.30 | 1.17 | 12.46 |
|  | 2007 |  | Exponential | 0.12 | 4.66 | 18.32 |
|  | 2008 |  | Spherical | 0.00 | 3.12 | 16.91 |
|  | 2009 |  | Spherical | 0.00 | 2.85 | 14.81 |
|  | 2010 |  | Spherical | 0.00 | 2.03 | 13.61 |

References: Morfin M, Fromentin J-M, Jadaud A, Bez N (2012) - *Spatio-Temporal Patterns of Key Exploited Marine Species in the Northwestern Mediterranean Sea*. PLoS ONE 7(5): e37907. doi:10.1371/journal.pone.0037907.
