# Supplementary material for: The Seascape of Demersal Fish Nursery Areas in the North Mediterranean Sea, a First Step Towards the Implementation of Spatial Planning for Trawl Fisheries
Source: PLoS One. 2015 Mar 18;10(3):e0119590. doi: 10.1371/journal.pone.0119590 (PMC4364973; doi:10.1371/journal.pone.0119590)
Supplement: S1 Text — (DOCX) [file pone.0119590.s003.docx]

**Text S1 FOR**

**The seascape of demersal fish nursery areas in the North Mediterranean Sea, a first step towards the implementation of spatial planning for trawl fisheries**

Francesco Colloca^1*^, Germana, Garofalo^1^, Isabella Bitetto^2^, Maria Teresa Facchini^2^, Fabio Grati^3^, Angela Martiradonna^2^, Gianluca Mastrantonio^4^, Nikolaos Nikolioudakis^5^, Francesc Ordinas^6^, Giuseppe Scarcella^3^, George Tserpes^5^, M. Pilar Tugores^6^, Vasilis Valavanis^5^, Roberto Carlucci^7^, Fabio Fiorentino^1^, Maria C. Follesa^8^, Magdalena Iglesias^6^, Leyla Knittweis^9^, Eugenia Lefkaditou^5^, Giuseppe Lembo^2^, Chiara Manfredi^10^, Enric Massutí^6^, Marie Louise Pace^11^, Nadia Papadopoulou^5^, Paolo Sartor^12^, Christopher J. Smith^5^, Maria Teresa Spedicato^2^

1 Istituto per l’Ambiente Marino Costiero (IAMC), Consiglio Nazionale delle Ricerche, Mazara del Vallo, Italy, 2 COISPA Tecnologia & Ricerca, Bari, Italy, 3 Istituto di Scienze Marine (ISMAR), Consiglio Nazionale delle Ricerche, Ancona, Italy, 4 Department of Economics, Roma Tre University, Italy, 5 Hellenic Centre for Marine Research, Greece, 6 Instituto Español de Oceanografía, Centre Oceanogràfic de les Balears, Spain, 7 Department of Biology, University of Bari, Italy, 8 Department of Life and Environmental Sciences, University of Cagliari, Italy, 9 Department of Biology, University of Malta, Malta, 10 Laboratorio Biologia Marina Fano, Italy, 11 Department of Fisheries and Aquaculture, Ministry for Sustainable Development, the Environment and Climate Change, Malta, 12 Centro Interuniversitario di Biologia Marina ed Ecologia Applicata “G. Bacci” (CIBM), Livorno, Italy.

**Cross validation indices (CVI) used for model selection**

**Section 1: Ordinary Kriging.**

The results of interpolations were tested with the cross-validation method. Cross-validation is based on excluding a value measured in a selected point and determining a new value in the same point, also taking into consideration the remaining observation. The procedure is repeated for all locations. The square root of the mean error (cross validation index) is then calculated as follows:

CVI=$\sqrt{\frac{1}{n}\sum_{i=1}^{n} {({Pred}_{i}-{Obs}_{i})}^{2}}$

where: ${Obs}_{i}$ = observed value in the point *i;* ${Pred}_{i}$*=* assessed value of the selected observation in the point *i*. R^2^ is the correlation index calculated between the observed values and the values predicted by cross-validation.

**Section 2: Generalized linear mixed models (GLMM) and Generalized additive mixed models (GAMM)**

In this case, the computation of the CVI was done selecting only m points among the n and the equation used was

CVI=$\sqrt{\sum_{i=1}^{m} \frac{{({Pred}_{i}-{Obs}_{i})}^{2}}{\sqrt{Var({Pred}_{i})}}}$

where $Var({Pred}_{i})$ is the variance of the prediction on point *i*.
